# Effects of Pesticide Mixtures and Environmental Factors on Benthic Diatom Communities: A Microcosm Approach

**DOI:** 10.1021/acs.est.5c14415

**Published:** 2026-04-20

**Authors:** Sarah Descloux, Ahmed Tlili, Alexandra Kroll, Soizic Morin, Christoph Schür, Kristin Schirmer, Nele Schuwirth

**Affiliations:** † Eawag, Swiss Federal Institute of Aquatic Science and Technology, Department of Environmental Toxicology (Utox), Überlandstrasse 133, 8600 Dübendorf, Switzerland; ‡ Eawag, Swiss Federal Institute of Aquatic Science and Technology, Department of Systems Analysis, Integrated Assessment and Modelling (Siam), Überlandstrasse 133, 8600 Dübendorf, Switzerland; § ETH Zürich, Department of Environmental Systems Science, Universitätstrasse 16, 8092 Zürich, Switzerland; ∥ Swiss Centre for Applied Ecotoxicology, Überlandstrasse 133, 8600 Dübendorf, Switzerland; # INRAE, National Research Institute for Agriculture, Food and the Environment, UR EABX, 50 Avenue de Verdun, 33612 Cestas Cedex, Nouvelle-Aquitaine Bordeaux Centre, France

**Keywords:** multifactorial experimental design, next-generation sequencing, agricultural pollutants, stress-response relationships, metabarcoding

## Abstract

Protecting aquatic ecosystems from pesticide pollution is a critical regulatory task. Benthic diatoms, being part of aquatic biofilms, are commonly used to indicate nutrient pollution. However, their potential as bioindicators for pesticides and other co-occurring environmental stressors remains poorly assessed. To address this, we conducted a flow-through chamber experiment designed to reflect realistic field exposure scenarios. We exposed biofilms to 44 treatments combining environmentally relevant pesticide mixtures including herbicides, fungicides, insecticides (44 levels of measured Toxic Units (TU_mix_) ranging from 2 × 10^–5^ to 4), in combination with three environmental stressors (light intensity, temperature, nutrient levels) over a 25-day period. Upon termination, we examined the diatom community composition using Next-Generation Sequencing along with community-level biofilm descriptors (β-diversity, effective photosynthetic activity, chlorophyll-a content, ash-free dry weight, bacterial abundance). While we did not find any significant responses to pesticides at the community level, we identified species-specific patterns of sensitivity or tolerance to pesticides and other experimental factors, thereby identifying them as potential bioindicators. These candidate species represent a first step toward a diatom-based index for pesticide stress assessment in Swiss streams. These findings highlight the need for realistic, multifactorial experiments to assess the impact of stressors in aquatic ecosystems.

## Graphical abstract

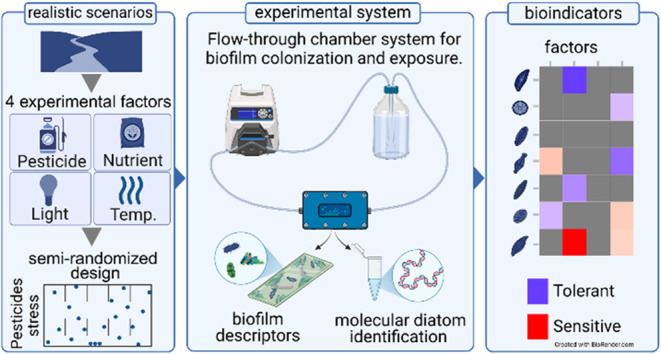

## Introduction

Agricultural herbicides have been the primary strategy for weed control for decades.^1^ These compounds frequently reach aquatic ecosystems, e.g., through surface water runoff and drift, impacting nontarget organisms such as freshwater microalgae, which, as primary producers, are crucial components of trophic networks.^2,3^ Consequently, efficient, and cost-effective assessment methods are vital for evaluating the ecological status of surface waters, especially for the detection of pesticide-related disturbance of aquatic communities, including microalgae.

Biomonitoring offers a rapid and comprehensive assessment of environmental stressors and chemical pollution in aquatic ecosystems and provides a time- and effect-integrated perspective, complementing chemical monitoring.^4^ In this context, benthic diatoms can effectively indicate a variety of natural and anthropogenic stressors, including organic pollution,^5^ metal pollution,^6^ eutrophication^7^ and acidification.^8^ Additionally, their short generation time, ability to colonise any submerged substrate, and ease of sampling,^9–11^ facilitate their wide use as bioindicators.^12^ Numerous in situ surveys demonstrate that diatom communities are affected by pesticide contamination, as shown by shifts in community composition^1,13–15^ and their physiological responses to pesticide stress.^3,16,17^ Moreover, diatom communities often exhibit stronger responses to these factors than to pesticide exposure, further complicating the assessment of pesticide-specific effects.^18^ In line with these findings, field studies frequently report that diatom diversity metrics track eutrophication more strongly than pesticide pressure, reflecting co-occurring stressors in agricultural catchments.^19,20^

Given the above, developing a bioassessment method for pesticides based on diatoms presents several challenges. Most importantly, pesticides are frequently present as mixtures rather than as single compounds.^1,21,22^ Therefore, diatom responses to complex pesticide pollution differ significantly depending on the combinations^23^ and concentrations of pesticides,^24^ species composition^25^ and testing setup,^26^ in addition to the environmental factors (light, nutrient levels, temperature).^14,27,28^ Adding to this complexity, anthropogenic stressors, such as nutrient and pesticide inputs from agriculture, often co-occur.^29^

Existing studies predominantly assess general responses such as biomass or growth, and analyses of community structure are often limited to broad taxonomic shifts.^13,30,31^ The influence of co-occurring stressors further complicates interpretation, leading to strong environmental context dependency and making it difficult to establish clear links between pesticide exposure and ecological responses.^32^ As a result, diatom responses to complex pesticide mixtures are still insufficiently characterized.^33^

Collectively, the difficulties in disentangling the role of factors influencing diatom community composition have thus far prevented the development of a diatom index that provides a causal link to pesticide exposure, and existing diatom-based indices primarily assess nutrient status rather than toxic contaminants such as pesticides.^34,35^

A pesticide-focused index for diatoms in Australian rivers was proposed by Wood et al. (2019)^20^ using the SPEAR (Species-At-Risk) framework. However, since it was derived from field data, it remains unclear how it is affected by co-occurring stressors, such as nutrients, and its direct transferability to other regions remains limited due to biogeographic differences in species pools.^36^ Another challenge in developing a diatom-based index lies in the taxonomic identification of species and the estimation of their relative abundances. While morphological techniques are well established and widely used for biomonitoring,^37^ they are time-consuming and require extensive training. As a promising tool, Next-Generation Sequencing (NGS) approaches that combine DNA barcoding^38^ and harness high-throughput sequencing to resolve species identification in community samples^39^ have emerged as a faster alternative for identifying diatom community composition in bulk samples.^40^

Indeed, targeted case studies have employed the ribulose-1,5-bisphosphate carboxylase/oxygenase large subunit gene (*rbcL, chloroplast gene*) primers^41^ that effectively discriminate among diatom species to profile community assemblages for ecological status assessments of water bodies. More recently, large-scale studies have shown that diatom *rbcL* metabarcoding displays strong, reproducible correlations with key water-chemistry parameters and can be used to derive indicator-taxon metrics suitable for biomonitoring.^42,43^ While it must be recognized that studies report discrepancies between NGS data and microscope-based assessments of diatom abundance and presence/absence in natural communities^44,45^ as well as in mock communities,^46^ NGS methods can facilitate efficient detection of global diatom molecular signals for comparison among different experimental conditions.

In this study, we assess the effects of pesticide pollution on benthic diatoms and the biofilm community under realistic conditions by conducting a flow-through chamber experiment with 44 treatments that combine pesticide mixtures with gradients of light, temperature and nutrients. Impacts on biofilm communities were assessed based on function (photosynthetic efficiency) and structure (chlorophyll-a content, ash-free dry weight, bacterial biomass), as well as by molecular amplicon sequencing to identify diatom species and estimate their relative abundances. Ultimately, this multifactorial microcosm experiment (i.e., simultaneously manipulating pesticides, light, temperature, and nutrients) aims to disentangle pesticide impacts from confounding factors, with the long-term objective of using diatoms as pesticide indicators.

## Materials and Methods

### Flow-Through Chamber System and Biofilm Colonisation

We designed a flow-through chamber system to allow for the growth of biofilm under controlled conditions. Biofilms were grown in this system under laminar flow conditions on glass substrates of 19.5 cm^2^ (75 × 26 mm^2^) facilitating biofilm adhesion and development on homogeneous surfaces (Supporting Information S1). This closed, recirculating setup allowed the application of independent treatments across 44 flow-through chambers, with flow velocity regulated at 9 mm/s by a multichannel peristaltic pump (Masterflex, Ismatec, United States) operating at maximum capacity. While this flow rate is not representative of natural watercourses, it still ensures a continuous flow that supports uniform biofilm development. The flow-through chambers were continuously supplied with medium from 1 L Schott bottles, each containing 750 mL of water from a stream in the vicinity of the lab (Chriesbach, 47°24′16.7″N, 8°36′41.4″E; Dübendorf, Switzerland). To ensure homogeneity, roughly 50 L of water were collected and pooled in a mixing tank before distributing aliquots into the bottles (Supporting Information S1 & S2). Water changes were performed twice a week to prevent pesticide and nutrient depletion and to avoid waste accumulation in the medium. The system was preconditioned for 4 days with the corresponding treatments before the start of the experiment to ensure constant biofilm exposure. Chemically inert silicone tubing (Maagtechnic, Switzerland) linked each Schott bottle to its flow-through chamber, and pressure-resistant two-stop tubing (Masterflex, Ismatec) connected the chambers to the peristaltic pump (Supporting Information S1). A lighting system simulating the sunlight spectrum (Philips Master LED tubes HF 1200 mm) provided a 12-h light/12-h dark cycle. After a 25-day colonisation period, glass slides were sampled and promptly processed for biological analyses.

### Experimental Design

Four experimental factors were investigated in combination (Figure 1): pesticide mixtures defined based on their toxic unit levels (TU; see section “Selection of Pesticide Mixtures” for more details), light intensity, temperature, and nutrient level. We employed a Latin Hypercube Design (LHD) to efficiently sample the four-dimensional experimental factor space.^47^ In an LHD, the ranges of the four dimensions are divided into equal segments, ensuring that each segment contains only one point (in this case, one sample). The four dimensions are then randomly combined. This strategy increases the statistical robustness of subsequent analyses. It facilitates a more comprehensive exploration of the multidimensional factor space compared to a full-factorial design with replicates, given the same number of experimental units.^47^ Treatments were randomly allocated to 44 flow-through chambers (IDs 1–44) using R (R Core Team, 2025). Temperature and light treatments were distributed in blocks owing to the limited number of temperature-controlled facilities capable of housing the microcosm setup, and the logistical challenge of installing more than two light sources per facility. For control chambers (IDs 17–20), which received no pesticide or nutrient additions, four replicates were maintained under intermediate temperature and light conditions (17.5 °C and 130 μmol photons m^–2^ s^–1^) to assess variability (see Supporting Information S3).

**Figure 1:**
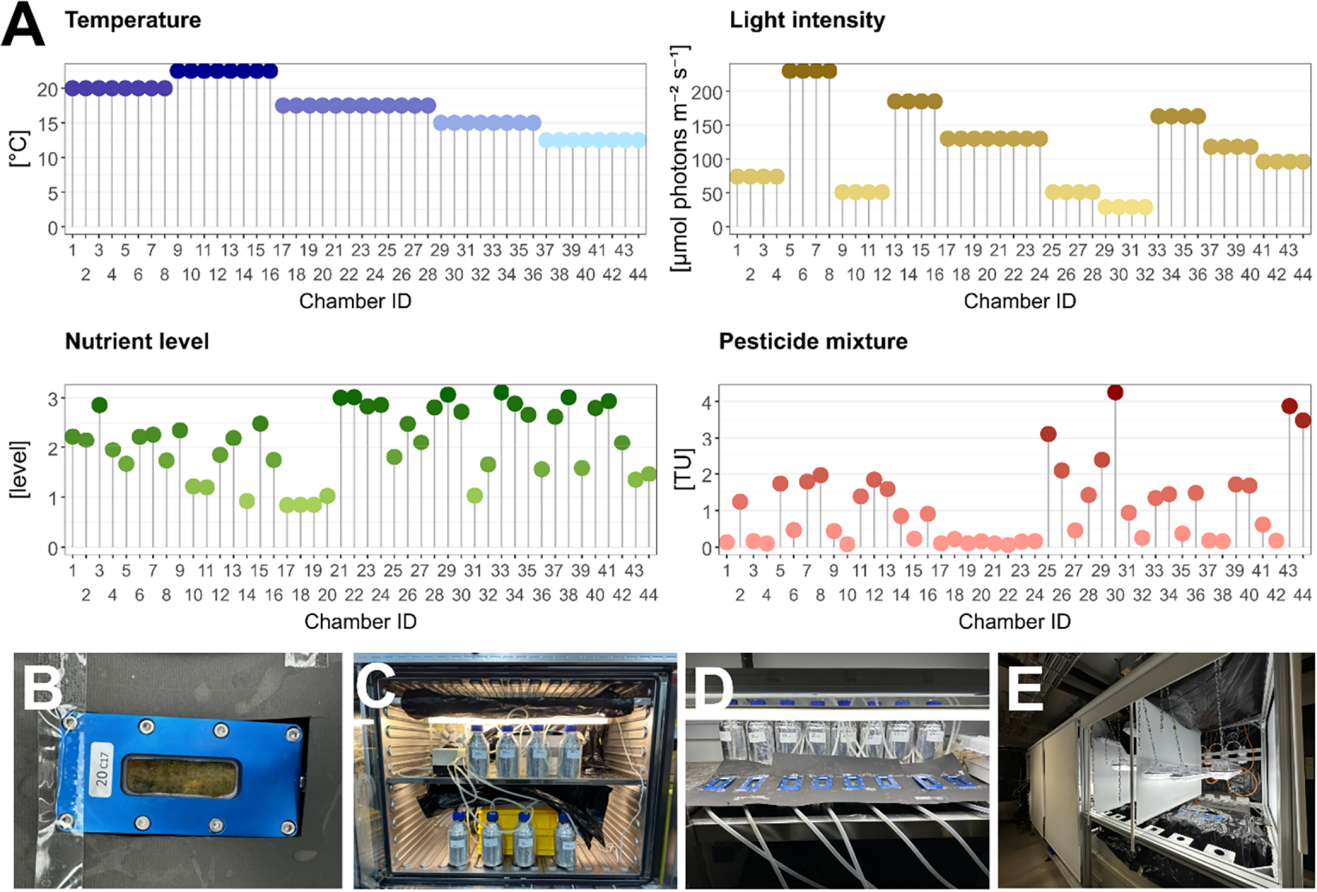
Experimental design and setup. (Panel A) shows the experimental design with a Latin Hypercube Design that accounts for technical constraints. It displays the distribution of four experimental factors: temperature, light intensity, nutrient level, and pesticide mixture, across 44 treatments. Temperature (top left) ranges from 12.5 to 22.5 °C. Light intensity (top right), measured in μmol photons m^–2^ s^–1^, ranges from 29 to 230 PAR. Nutrient level (bottom left) ranges from 0.84 to 3.11. Pesticide mixture (bottom right) ranges from 2 × 10^–5^ to 4 Toxic Units. All parameters are based on measured values. (Panel B) shows a flow-through chamber colonised by biofilm after 19 days. (Panel C) displays eight treatments maintained at 12.5 °C in an incubator. (Panel D) shows eight treatments at 17.5 °C in a cold room, where chambers and tubing are covered to avoid external contamination. (Panel E) presents the setup in a facility, which hosts treatments at 20 and 22.5 °C.

### Experimental Factors

#### Selection of Pesticide Mixtures

The goal of this study was to conduct an experiment that closely reflects the actual pesticide conditions in Swiss streams. Therefore, we considered 49 pesticides that are monitored and regularly detected by the Swiss National Surface Water Quality Monitoring micropollutants NAWA MP^48^ (extension of NAWA TREND, NAWA – www.bafu.admin.ch, program during the years 2018 and 2019). The selected pesticides comprise 24 herbicides, 12 fungicides, and 13 insecticides, all of which were authorized for use in Switzerland in 2018–2019 and frequently exceeded environmental quality standards (EQS) for freshwater ecosystems, posing a significant risk to aquatic organisms.^49^

To evaluate the expected toxicity of a pesticide mixture, we applied the concept of Toxic Units (TU),^50,51^ which is defined as the sum of the ratios of substance concentrations to their estimated toxicity threshold

1 $$t\mathrm{oxic}\;u\mathrm{nit}\;\left(TU\right)=\sum_{i}\frac{c\mathrm{oncentration of}\;s\mathrm{ubstance}\;i}{t\mathrm{oxicity of}\;s\mathrm{ubstance}\;i}$$

The toxicity threshold values were derived from the lowest available relevant and reliable effective concentration 50 (*EC*_50_) for primary producers, obtained from the Ecotox Centre database (Supporting Information S4).

The TU for all samples monitored under the NAWA MP program (denoted as TU_sample_) was calculated using the script provided in the study by Schuwirth (2020).^52^ For each TU targeted in our experiment (TU_target_), a corresponding NAWA sample exhibiting a similar TU was identified. To select appropriate samples, a probabilistic method was applied, giving higher probabilities to those with TU values (TU_sample_) closest to the targeted TU (TU_target_). Once selected, the concentrations of the substances measured in the sample were proportionally adjusted to precisely match the desired TU_target_. A detailed description of this method is available in the Supporting Information S4.

#### Selection of Nutrient Concentrations

The range of nutrients, representing realistic scenarios for Swiss surface waters, was defined based on the Swiss assessment method for nutrients in surface waters from the Federal Office of the Environment (FOEN), which provides indicator values for chemical parameters using a 5-category scale (Liechti, 2010, Supporting Information S5). The experimental medium (i.e., streamwater from Chriesbach) was supplemented with nitrate (NO_3_^–^, ranging from 4 to 22.4 mgN/L), phosphate (PO_4_^3–^, ranging from 0.06 to 0.32 mgP/L), silicic acid (H_4_SiO_4_, ranging from 4 to 16 mgSi/L) and dissolved organic carbon (CH_3_COONa, ranging from 2.5 to 16 mgC/L). The lowest concentrations (control treatment) correspond to those of the Chriesbach water, which, based on previous measurements over 12 months in 2020, were expected to be approximately 4 mg N/L for nitrate (good water-quality class), 0.06 mg P/L for phosphate (poor water-quality class), 2.5 mg C/L for dissolved organic carbon (good water-quality class), and 4 mg Si/L for silicic acid (neither regulated nor classified). Because the exact concentrations in the Chriesbach water were unknown at the start time of the experiment, we estimated the spike volumes required to achieve our target concentrations based on these prior measurements. In the following, we represent the results based on measured concentrations.

#### Selection of Exposure Temperatures

A total of five temperatures were used, each maintained in a separate controlled environment: 12.5 °C in one incubator, 15 and 17.5 °C in two cold rooms, and 20 and 22.5 °C in two temperature-controlled tanks. This temperature range corresponds to measured water temperatures of Swiss rivers (FOEN - Temperatures of watercourses) and falls within the thermal tolerance of freshwater diatoms, mirroring the conditions typically employed in algal-growth experiments.^54,55^

#### Selection of Light Intensities

A total of nine light intensities (29, 51, 74, 96, 118, 130, 163, 185, and 230 μmol photons m^–2^ s^–1^) was set, with two intensities assigned per incubator. They lie within environmentally relevant and experimental benchmarks: spring and fall irradiance in forest stream often reach or exceed 200 μmol photons m^–2^ s^–1^.^56^ They also represent both the typical light conditions used in algal experiments^57^ and the range that was technically feasible within the constraints of the setup.

### Water Sampling and Chemical Analysis

We collected water samples for nutrient and pesticide analyses from all 44 treatments twice weekly: immediately after adding pesticides and nutrients and again after 3–4 days of exposure, just before each media change. Samples for nutrient analysis were filtered using 0.45 μm cellulose nitrate membrane filters (Sartorius) prior to being stored in 250 mL HD polyethylene bottles (Nalgene) at 4 °C until analysis. Pesticide samples were taken in duplicates and stored in 3 mL dark vials (Supelco) at −20 °C to prevent degradation. In parallel, pH, conductivity, and oxygen concentration were measured at the start and end of the experiment, as well as before and after two water changes, using a portable multiparameter meter (WTW Meters, Germany).

Pesticide concentrations (49 compounds) were analyzed using High Performance Liquid Chromatography coupled with Mass Spectrometry (HPLC-MS/MS). The system consisted of a PAL HTS-xt Autosampler (CTC Analytics, Switzerland), an UltiMate 3000 HPLC pump (Thermo Fisher Scientific, USA), and an Atlantis T3 column (3 × 150 mm^2^, 3 μm particle size; Waters), based on Kiefer et al. (2021)^58^ with few modifications. For the measurement, 100 μL of sample was injected at a flow rate of 300 μL/min, with the column maintained at 30 °C. Mass detection was performed using an LTQ Orbitrap XL (Thermo Fisher Scientific), operating at a resolution of *R* = 140,000 at *m*/*z* 400. The system used Electrospray Ionization (ESI) in both positive and negative modes, with spray voltages set at +4 kV and −4 kV, and a capillary temperature of 325 °C. Initially, data-dependent acquisition was applied with six MS/MS scans (1.5 Da isolation window) following each full scan. The samples were later reanalyzed using a Q Exactive Orbitrap Mass Spectrometer (Thermo Fisher Scientific) under a similar setup, including targeted MS/MS and inclusion lists for improved data identification. All high-resolution mass spectrometry data were processed using Skyline software (MacCoss Lab, University of Washington) (Supporting Information S6).

### Biofilm Collection and Characterization

Biofilm from each flow-through chamber was scraped off and suspended in 10 mL of Evian mineral water to ensure homogenization while minimizing the impact of water minerality on the organisms.^59^ From this suspension, 2 mL were taken for diatom NGS and stored at −20 °C until further processing. The remaining volume was directly used for functional and structural biofilm characterization, including measurements of ash-free dry weight (AFDW), chlorophyll-a content, photosynthetic efficiency, and bacterial biomass.

Total biomass was determined as AFDW following the method described by Tlili et al. (2008).^26^ In brief, 2 mL of biofilm suspension were vacuum filtered onto glass fiber filters (Whatman), weighed, dried at 60 °C for 24 h prior to reweighing, combusted at 450 °C for 1 h, and reweighed to calculate the organic matter lost.

Chlorophyll-a content, serving as a proxy for algal biomass, was measured following Sartory & Grobbelaar (1984).^60^ Two mL were filtered onto Whatman filters, which were extracted in 90% acetone for 24 h in the dark, and fluorescence was quantified on a UV/vis spectrophotometer (Cary100) at 665–700 nm. Chlorophyll-a concentrations were then normalized to AFDW.

Photosynthetic efficiency was assessed using an Imaging-PAM fluorimeter (MAXI-I-PAM, Heinz Walz GmbH, Germany). Aliquots (0.5 mL) of homogenized biofilm suspension were dispensed into a 24-well microplate and measured at room temperature without prior dark adaptation. Samples were exposed to a standardized actinic irradiance of 81 μmol photons m^–2^ s^–1^ (verified to be within the detectable, nonlimiting, and nonsaturating range) for approximately 8 min. A saturating pulse (2800 μmol photons m^–2^ s^–1^) was then applied to determine maximum fluorescence under actinic light (F′m). Chlorophyll-a fluorescence was excited at 650 nm, and the effective PSII quantum yield was calculated as

2 $$\Phi \mathrm{PSII}=\frac{F^{\prime }m-F^{\prime }}{F^{\prime }m}$$

Standardised scraping and homogenization across all samples allowed photosynthetic efficiency to be compared among treatments.

Bacterial biomass was estimated following Frossard et al. (2012)^61^ with slight modifications. Basically, bacterial abundance was quantified by fixing 3 mL of biofilm suspension in an equal volume of 4% phosphate-buffered formalin (can be stored at 4 °C for a maximum of one month), sonicating the mixture on ice to disperse cells, and clearing debris by two rounds of 2000 g centrifugation at 4 °C for 15 min. The recovered supernatant was stained with SYBR Green (100×) for 15 min in the dark, diluted 1:10 in Milli-Q water, spiked with a known concentration of fluorescent standard beads, and then run on a Gallios flow cytometer (Beckman Coulter); the ratio of bacterial events to bead events provided an absolute cell count.

### NGS of the Diatom ***rbcL*** Gene and Eukaryote (***18S***) and Prokaryote (***16S***) Genes

To assess the diversity of diatoms within the biofilm, other eukaryotes (e.g., green algae) and prokaryotes (e.g., bacteria and cyanobacteria), total genomic DNA was extracted from 2 mL aliquots of each biofilm suspension collected at the end of the exposure phase. DNA extraction was performed using the PowerBiofilm DNA Isolation Kit (MO BIO Laboratories), following the manufacturer’s protocol. Extracted DNA was quantified using a Qubit 1.0 fluorometer with the dsDNA HS Assay. A negative extraction control was included to test for contamination. Data produced and analyzed in this paper were generated in collaboration with the Genetic Diversity Centre (GDC), ETH Zürich (Swiss Federal Institute of Technology Zurich), Switzerland. Library preparation involved a two-step PCR targeting the *rbcL* gene with two distinct primer sets, as described in Herlemann et al. (2011).^62^ Triplicate PCRs were conducted per sample, alongside positive controls consisting of mock communities of 27 species (each at 100 ng/50 μL) (Supporting Information S7) for the *rbcL* and *18S* as well as a mock community from the GDC, consisting of 10 species, and negative controls for the *16S*. Sequencing was performed on an Illumina MiSeq platform (2 × 300 bp) using the MiSeq Reagent Kit v3. Primary processing and demultiplexing were done with MiSeq Control Software v2.2.

Paired-end amplicon sequences were processed and analyzed using a standardized pipeline based on USEARCH v11.0.667. Amplicon sequence variants were inferred using the UNOISE3 algorithm, resulting in zero-radius operational taxonomic units (ZOTUs). Taxonomic assignment was performed using the SINTAX classifier against the INRAE *rbcL* reference database (version 9.2, Diat.barcode), applying a confidence threshold of 0.85. For detailed parameters on read trimming, see Supporting Information S8. Raw sequencing data are available in the NCBI Sequence database under BioProject accession number PRJNA1337291.

### Data Analysis of Biofilm Descriptors and Diatom Community Composition

The significance of the effect of environmental factors (light, temperature, nutrients, and pesticide stress) on biofilm descriptors (i.e., AFDW, chlorophyll-a content, bacterial biomass, effective photosynthetic activity) was assessed in R (version 4.4.30, R Core Team, 2025).^63^ Pairplots were generated using the R package GGally (version 2.2.1)^64^ with a significance threshold of *p* = 0.05. Sequencing data analyses were performed with the R package Phyloseq (version 1.50.0).^65^ α-diversity (i.e., richness and evenness of a given biofilm community) was evaluated for each treatment via the Shannon and Simpson diversity indices and Chao1 species richness using the R package Phyloseq (version 1.50.0). The analysis of β-diversity (i.e., measuring the structural differences among communities) was based on Bray–Curtis distances, which use dissimilarity based on the relative abundances of taxa between samples. Permutational Multivariate Analysis of Variance (PERMANOVA) tests were carried out on the Bray–Curtis distances matrix using the R package vegan (version 2.6.10).^66^ Bray–Curtis distances were visualized using nonmetric multidimensional scaling (NMDS) using the R package ggplot2 (version 3.5.2)^67^ to illustrate sample clustering according to treatment conditions. Moreover, to complement the β-diversity analysis, PERMANOVA was performed on Jaccard and cosine dissimilarity matrices, and a Hellinger-transformed redundancy analysis (RDA) was used as an additional ordination approach (Supporting Information S12). To identify pesticide-indicative species, we used beta regression in R with the betareg package (version 3.2.3)^68^ to model the relative abundance of each species (a value constrained between 0 and 1) as a function of treatment factors (temperature, light, nutrients, and pesticide stress). Species present in at least 15 of our 44 samples were retained for analysis, ensuring robust statistical modeling, and enhancing the biomonitoring relevance of species-level responses. This threshold follows the recommendation of Peruzzi et al. (1996)^69^ to use more than ten events per variable. The regression coefficients for each species and factor were summarized in a heatmap, where positive and negative coefficients indicate the direction and magnitude of each species’ response to the treatments (Figure 3).

Interpreting community shifts from NGS read counts requires caution, since inter- and intraspecific variation in *rbcL* copy number can lead to the over- or underrepresentation of certain species. To account for these biases, Vasselon et al. (2018)^70^ proposed biovolume-based correction factors for eight diatoms using the *rbcL* primers. However, because our samples included many taxa, generating calibration curves for each species was beyond the scope of this study and therefore, we did not apply biovolume corrections. Although beta regression applied in the present study accounts for systematic, interspecific biases (e.g., DNA extraction efficiency, *rbcL* PCR amplification, sequencing depth, primer–reference mismatches, and database limitations), intraspecific copy-number variation remains an unavoidable source of uncertainty when interpreting relative-abundance estimates.

## Results

### Stability of Physicochemical Parameters Across Treatments

Conductivity, dissolved oxygen, and pH remained stable across all 44 treatments (Supporting Information S9). This applies to measurements that were taken immediately after the initial media change (day 1), during and at the end of the exposure periods (on day 5 and on day 25).

### Accuracy and Temporal Dynamics of Pesticide and Nutrient Exposure

The comparison of measured concentrations and the target values from the experimental design shows that for pesticides, initial pesticide concentration measurements for 44 different complex mixtures (containing 2 to 23 pesticides each) mainly cluster around the 1:1 line (Figure 2A), indicating accurate or marginally elevated dosing. The pesticide stress in chambers 25, 30, 43, and 44 measured just after the water change renewal lay above the 1:1 line. The most extreme measure was for diuron and metazachlor, which was present at twice the expected concentration. Most pesticide concentrations measured before each medium renewal (i.e., 3–4 days of exposure) fell well below the diagonal (Figure 2B), reflecting substantial loss. Nutrient levels (dissolved organic carbon (DOC), nitrate, phosphate, and silicic acid) show similar patterns (Figure 2C, D), with values that are higher than target values before the exposure (especially for the low target concentrations) and values that are mostly lower than target values after exposure (especially for the high target concentrations).

**Figure 2:**
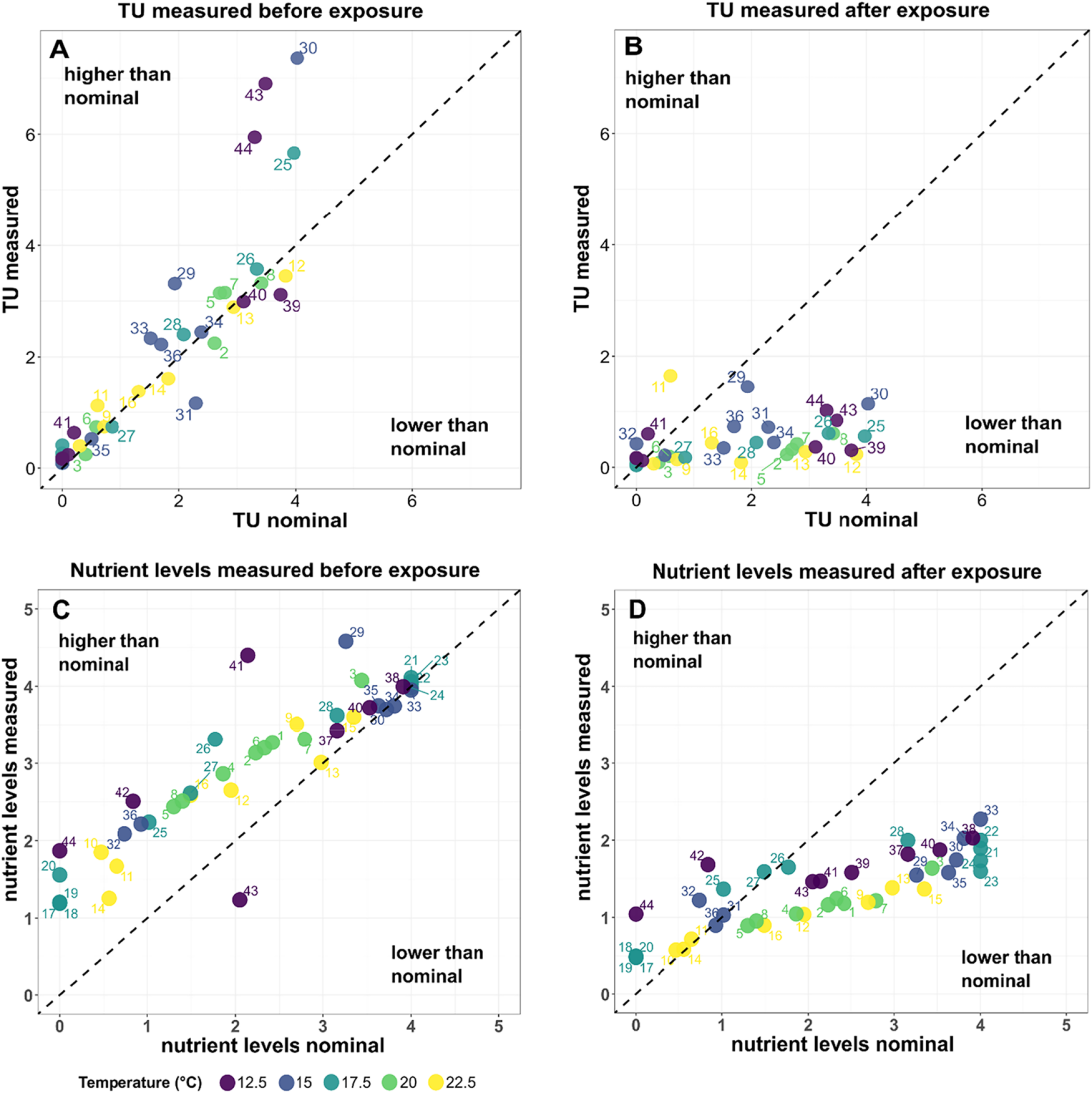
Comparison of nominal versus measured values for Toxic Units (TU, pesticide stress) and nutrient levels across 44 treatments quantified before (Panel A & C) and after (Panel B & D) exposure. The dashed diagonal in each plot denotes the 1:1 line (measured = nominal).

DOC levels declined after 3 to 4 days of exposure, approaching concentrations found in the Chriesbach water. Both phosphate and silicic acid showed a temperature-dependent loss pattern. This effect was especially pronounced in chambers 1 to 16, where higher temperatures (20 and 22.5 °C) appear to have enhanced nutrient use. Nitrate concentrations were rather stable and sometimes even increased over time (Supporting Information S10).

### Species-Specific Responses of Diatoms to Pesticide and Environmental Gradients

We were able to assign 66% of the zero-radius operational taxonomic units (ZOTUs; exact sequence variants) to the species level. A total of 50 diatom species were identified by *rbcL* gene sequencing, with over 95% of bases achieving a Phred score ≥ 30 (95% of the sequence data has an error probability of ≤ 1 in 1000 bases, indicating excellent sequencing quality) after quality filtering. The diatom diversity is representative of the regional diatom flora in Switzerland.^71^

Diatom species abundances in response to experimental factors were analyzed using beta regression to identify patterns of sensitivity and tolerance. A total of 23 species present in at least 15 of our 44 samples were retained for analysis, ensuring robust statistical modeling, and enhancing the biomonitoring relevance of species-level responses. The heatmap (Figure 3) shows significant correlations between species-level relative abundances and four experimental factors where several taxa exhibited clear directional responses. Temperature significantly impacted eight species, eliciting the strongest positive response in *Melosira varians* and the strongest negative response in *Achnanthidium minutissimum*, indicating contrasting thermal preferences. Light had a significant effect on ten species, followed by pesticide stress affecting seven species, and nutrient increase influencing three species (*Nitzschia palea*, *Planothidium frequentissimum*, *Sellaphora saugerresii*). These species-level responses further support community-level results, where temperature and light emerged as the main drivers of diatom community responses, largely through their pronounced effects on a few key species. *Amphora pediculus*, *Fragilaria gracilis*, *Planothidium frequentissimum*, *Planothidium victori* and *Staurosira venter* were positively associated with increasing pesticide stress levels, suggesting higher tolerance to pesticide stress. In contrast, *Fistulifera saprophila* and *Nitzschia amphibia* showed negative correlations with pesticide stress, indicating sensitivity.

**Figure 3:**
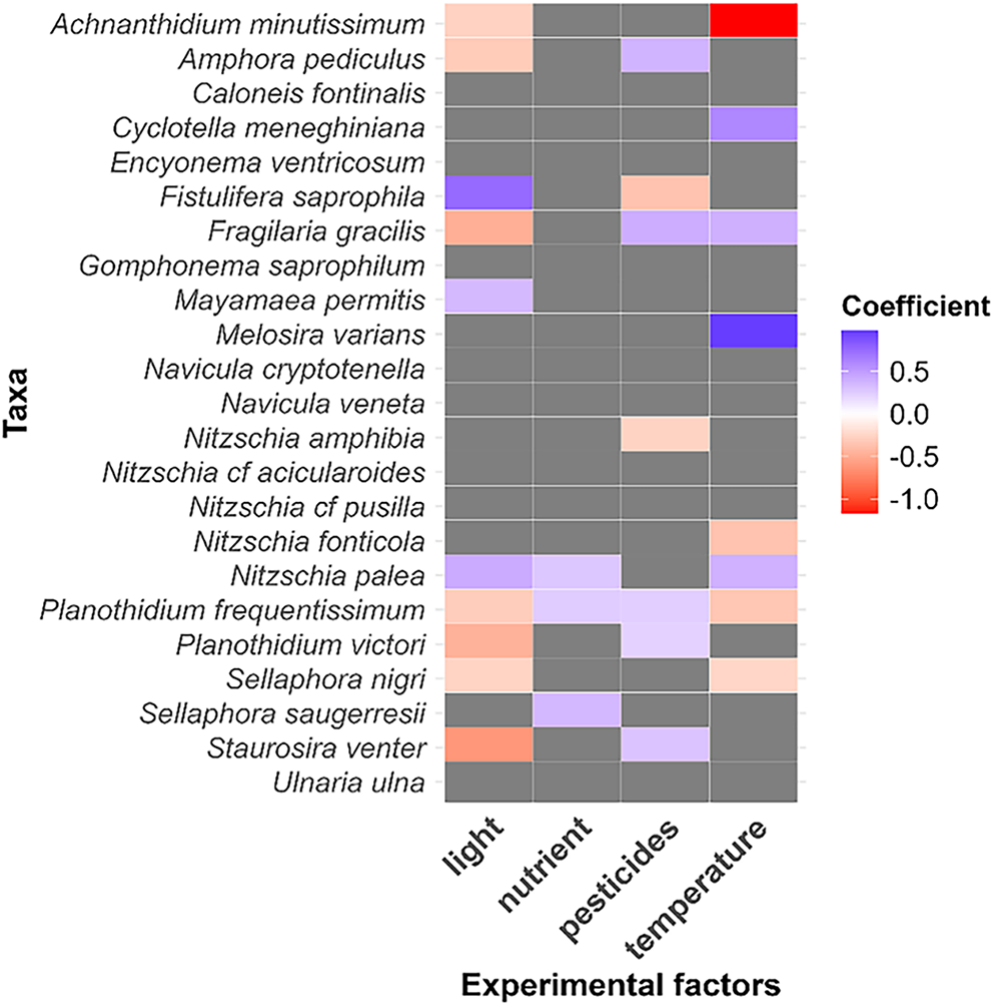
Heatmap of beta regression coefficients showing diatom species’ responses to experimental factors. Each row represents a species, and each column corresponds to one experimental variable. Shades of red indicate positive correlations, while blue shades indicate negative correlations with the corresponding factor. Gray cells denote nonsignificant relationships (*p* > 0.05). Coefficients near 0 reflect weak responses; values between ± 0.2 and ± 0.5 indicate moderate responses, and those beyond ± 0.5 represent strong responses. The 23 species shown are those that were present in at least 15 samples and thus retained for beta regression analysis.

### Consistent Alpha Diversity and Compositional Turnover of Diatom Communities

A four-way ANOVA indicated that Shannon diversity (as a measure of α-diversity) did not differ significantly with light (*p* = 0.08), temperature (*p* = 0.44), nutrient level (*p* = 0.13), or pesticide stress (*p* = 0.73). Conversely, β-diversity analyses revealed strong compositional structuring: a PERMANOVA based on Bray–Curtis dissimilarity indicated that temperature and light significantly influenced community composition, whereas nutrients and pesticide exposure had no significant effect (Figure 4). This relationship was consistent across additional distance- and variance-based analyses Jaccard and cosine PERMANOVA; Hellinger-based RDA (Supporting Information S12).

**Figure 4:**
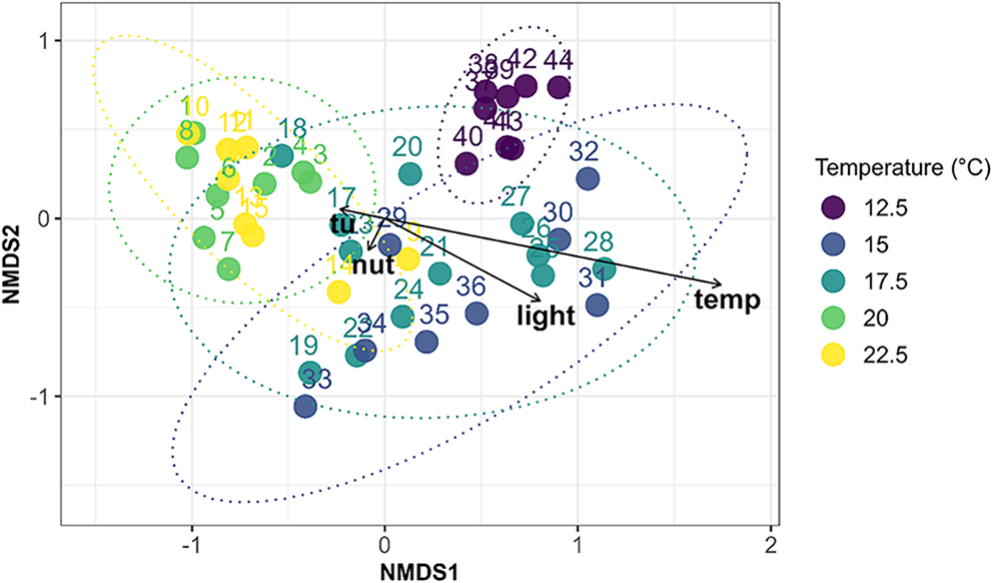
NMDS ordination (k = 2, stress = 0.12) of diatom community composition based on Bray–Curtis dissimilarity. Each point represents a sample, colored according to its temperature treatment (°C), with 95% confidence ellipses around treatment centroids. PERMANOVA on Bray–Curtis distances showed significant effects of temperature (*p* = 0.001) and light (*p* = 0.001), whereas nutrient (*p* = 0.16) and pesticide stress (*p* = 0.55) gradients were not significant. Environmental vectors from envfit are overlaid to indicate the direction and relative strength of each experimental factor.

The four control replicates (IDs 17–20) clustered near the center of the nonmetric multidimensional scaling (NMDS) ordination (Figure 4), falling within the overlapping 95% confidence ellipses of the midtemperature (17.5 °C) treatments, and confirming their consistency beyond natural community variability. The NMDS indicated temperature, followed by light, as the dominant gradient structuring the assemblages. Pairwise PERMANOVA further revealed that the community composition at 12.5 °C differed significantly from that observed at all higher temperatures (15, 17.5, 20, and 22.5 °C)

### Shifts in Relative Abundance of Dominant Diatom Taxa Across Treatments

The relative abundance of the ten most dominant diatom species varied considerably across the 44 samples (Figure 5). *M. varians* was the most abundant species of the community in 14 out of the 16 samples exposed to higher temperatures (i.e., chamber IDs 1–16 at 20 and 22.5 °C). In contrast, *A. minutissimum* comprised over 50% of the community in all eight samples exposed to the lowest temperature (chamber IDs 37–44 at 12.5 °C). Several samples at intermediate temperatures (e.g., chamber IDs 27–36 at 15 and 17.5 °C) exhibited more even community structures, with no species exceeding 50% the community. The “other” category, which includes all species apart from the top 10 (See Supporting Information S11), did not exceed 20% except for three samples (chamber IDs 28, 31, 32), indicating low abundances of other taxa.

**Figure 5:**
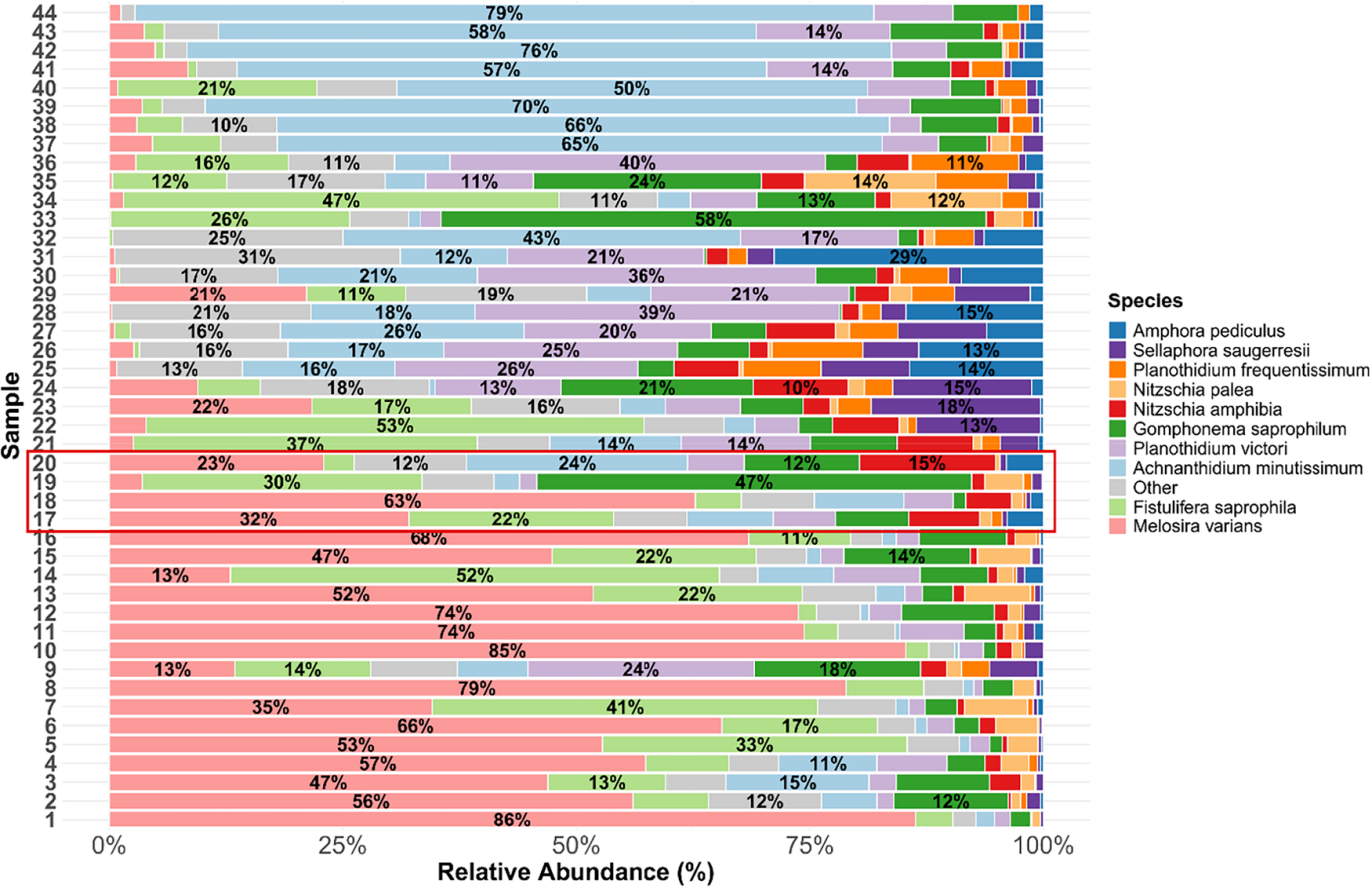
Relative abundance of the top 10 most abundant diatom species across 44 samples. Each bar represents one sample, with species-level relative abundances. All other species were grouped under “other”. The values in the bars are the percentages. The four control treatments (chamber IDs 17–20) are highlighted by a red box.

### Effect of Experimental Factors on Photosynthetic Efficiency, Chlorophyll-a Content, Ash-Free Dry Weight and Bacterial Biomass

Correlation analysis of experimental factors on biofilm structure and function revealed that only light intensity had a significant effect, correlating positively with bacterial abundance (Figure 6). Among the response variables, two significant inter-relationships were observed: ash-free dry weight (AFDW) was negatively correlated with chlorophyll-a content and positively correlated with bacterial abundance. Four treatments at the lowest light intensity of 29 μmol photons m^–2^ s^–1^ (chamber IDs 28–31) and one treatment at 51 μmol photons m^–2^ s^–1^ (chamber ID 32) did not develop sufficient biofilm for the assessment of biological parameters, reducing the number of assessable treatments to 39 and indicating an effect of light on biomass, even though it could not be quantified.

**Figure 6:**
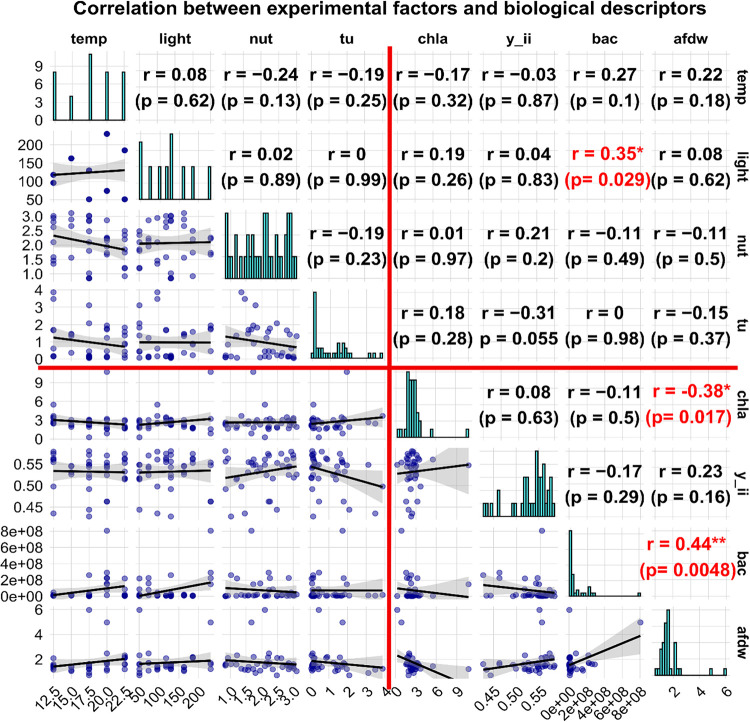
Correlogram illustrating the pairwise relationships between the four experimental factors: temperature (temp), light intensity (light), pesticide stress (tu), and nutrient levels (nut), with the biological descriptors: chlorophyll-a content (chla), effective photosynthetic efficiency (y_ii), bacterial abundance (bac), and ash-free dry weight (afdw) across treatments. The diagonal cells show histograms for each variable distributions, while off-diagonal cells contain scatterplots visualizing the relationship between two variables. In the upper triangular cells, correlation coefficients (r values) quantify these relationships, ranging from −1 to 1, where values closer to 1 indicate a strong positive correlation, values closer to −1 indicate a strong negative correlation, and values near 0 suggest little to no linear relationship. The red cross marks the upper-right region of the correlogram, which shows the correlation coefficients between the four experimental factors (temp, light, tu, nut) and the four biological descriptors (chla, y_ii, bac, afdw). Asterisks beside the correlation coefficients denote statistical significance, with one star (*) for *p* < 0.05, two stars (**) for *p* < 0.01, and three stars (***) for *p* < 0.001. Each axis shows the specific scale for each environmental or biological parameter: temp ranges from 12.5 to 22.5 °C, light from 51 to 230 PAR, tu from 2 × 10^–5^ to 4, nut from 0.84 to 3.11.

Control replicates (chamber IDs 17–20) showed small variability with a coefficient of variation (CV ≈ 3.7%) in AFDW and chlorophyll-a (CV ≈ 7.5%), and moderate variability in photosynthetic efficiency (CV ≈ 13.3%). However, bacterial abundance was highly variable (CV ≈ 193%), driven by ID 20 (260 × 10^6^ cells mL^–1^ versus 2.02, 2.77, and 1.72 × 10^6^ cells mL^–1^ in chamber IDs 17–19).

### Community Responses of Prokaryote and Nondiatom Eukaryote Communities to Experimental Factors

For prokaryotes (based on *16S* analysis) assigned at the phylum level, Shannon diversity did not vary significantly among treatments (light: *p* = 0.08; temperature: *p* = 0.29; nutrients: *p* = 0.31; pesticide stress: *p* = 0.98). Bray–Curtis dissimilarity of prokaryotes differed significantly only with temperature (*p* = 0.001), whereas light (*p* = 0.13), nutrient concentration (*p* = 0.19) and pesticide exposure (*p* = 0.61) had no significant effect. An NMDS ordination illustrating these patterns is provided in the Supporting Information S13. A multiple-linear regression of absolute cyanobacterial counts against the four experimental factors revealed that only temperature had a significant positive effect (*p* = 0.04). Neither light intensity (*p* = 0.66), nutrient concentration (*p* = 0.21), nor TU exposure (*p* = 0.51) explained a significant fraction of the variation in cyanobacterial counts.

For eukaryotes based on 18S and assigned at the genus level, Shannon diversity decreased with increasing temperature (Spearman’s ρ = −0.4, ANOVA *p* = 0.05), while no significant effects were detected for light intensity (*p* = 0.6), nutrient concentration (*p* = 0.3), or pesticide exposure (*p* = 0.7). PERMANOVA on Bray–Curtis dissimilarity indicated that eukaryote community composition varied significantly with light (*p* = 0.017), and temperature (*p* = 0.001), while neither nutrient concentration (*p* = 0.2) nor pesticide stress exposure (*p* = 0.2) had a significant effect (Supporting Information S14). Spearman correlation analyses further identified *Melosira*, *Achnanthidium*, and *Planothidium* as the genera most strongly associated with the light and temperature gradients (*p* < 0.05).

## Discussion

### Pesticide and Nutrient Loss during Exposure

While the measured pesticide and nutrient concentrations closely matched target concentrations prior to exposure, we observed a loss during the exposure period despite the biweekly renewal of the medium. Minor deviations from the target concentration prior to exposure likely arose from extensive handling of multiple compounds at low concentrations, e.g., sample 30 required mixing 12 pesticides at concentrations ranging from 4 × 10^–4^ μg/L to 3.6 μg/L. We considered five processes as potential contributors to pesticide loss during the exposure period between the biweekly renewals. (1) Hydrolysis: Based on the hydrolysis half-lives of the pesticides (Pesticides Properties DataBase, accessed 18 September 2025), the expected loss to hydrolysis over a 3–4-day exposure time is insufficient to explain the observed decline (Supporting Information S6). (2) Sorption: We rigorously preconditioned all tubing with the corresponding treatment to minimize sorption losses to system components.^72,73^ Nevertheless, given the large area of wetted surfaces (particularly the ≈ 2.5 m of silicone tubing per treatment), it is possible that sorption contributed to the observed loss. In particular, concentrations of several pesticides with a high log Kow that are expected to sorb to silicone tubing and biofilm, decreased substantially. Examples are chlorpyrifos (log Kow = 4.7; median loss 88%) and terbuthylazine (log Kow = 3.4; median loss 98%).^74^ (3) Abiotic degradation: other than hydrolysis, temperature and light could enhance the degradation of pesticides parent compounds.^75^ However, we do not see a significant temperature or light impact in the deviations from the target values. (4) Biotic degradation and bioaccumulation: microbial transformation of pesticides (e.g., iprovalicarb and mecoprop) within the biofilm matrix can lead to a loss, and some compounds may also be bioaccumulated in biofilms (e.g., isoproturon) as reported by Desiante et al. (2021)^74^ and Kaur et al. (2023).^75^ (5) Losses to headspace: since the Henry’s-law constants of the pesticides tested are lower than that of water, important volatilisation into the bottle headspace is unlikely. Overall, sorption and biotic degradation appear to be the most plausible contributors to the reduction in pesticide concentrations observed after 3–4 days of exposure.

Nutrient concentrations at the administration time were generally higher than the targeted values, which we associate with the background levels of nutrients in the Chriesbach stream (the actual concentrations in Chriesbach were 8 mg C/L DOC, 8 mg P/L PO_4_^3–^, 15 mg Si/L H_4_SiO_4_ and 5 mg N/L NO_3_^–^, while we expected on average 2.5 mg C/L DOC, 6 mg P/L PO_4_^3–^, 4 mg Si/L H_4_SiO_4_ and 4 mg N/L NO_3_^–^; see control treatments IDs 17–20). This dependence on the variability of nutrient concentrations cannot be avoided when using streamwater, which was important to allow for a natural colonisation of the biofilms. The loss of nutrients during exposure is likely due to their role as energy and nutrient sources for microbial communities. These communities can also rely on intracellular storage pools, such as polyphosphate granules in bacteria or intracellular silica storage in diatoms that contributes to frustule formation that reinforce nutrient uptake.^76^ While DOC can be utilized by heterotrophic bacteria and fungi within biofilms as a carbon and energy source,^77^ the complete loss observed in this experiment suggests that additional chemical transformation (e.g., extracellular enzymatic hydrolysis) might have played a role.^78^ Phosphate and silicic acid concentrations decreased more strongly at higher temperatures, likely reflecting enhanced microbial activity and stimulated diatom growth, thereby increasing nutrient consumption.^79,80^ In contrast, nitrate concentrations remained relatively stable over the same period consistent with the Redfield stoichiometry (N/P ≈ 16:1).^81^ In addition, nitrate is stable under various environmental conditions.^82,83^

### Pesticide and Experimental Factors Effects on Diatom Community Composition and Identification of Bioindicator Species

As illustrated by the beta regression results, several diatom species exhibited significant change in their relative abundance across chemical and abiotic gradients, identifying them as candidate bioindicators for biomonitoring: *A. pediculus* and *P. victori*, both known to tolerate herbicides, such as triazines (photosystem II inhibitor) and S-metolachlor (very-long-chain fatty acid (VLCFA) synthesis inhibitor),^2,84^ exhibited significant increase in their relative abundance to pesticide treatments. Similarly, *P. frequentissimum* showed an increase in relative abundance under pesticide stress, consistent with its reported tolerance to metolachlor in periphyton communities by Roubeix et al. (2011).^85^ *P. frequentissimum* relative abundance also increased under nutrient exposure, consistent with reports identifying this taxon as an indicator of phosphorus and nitrogen in freshwater ecosystems.^86^ *N. amphibia* exhibited a decrease in relative abundance under pesticide stress, a pattern not consistent with its previously reported tolerance to alachlor in biofilm communities.^87^ The relative abundance of *M. varians* increased significantly with temperature, confirming that *M. varians* is tolerant to high temperature.^55^ The significant decrease in relative abundance of *A. minutissimum* in response to temperature also aligns with previous findings by Delgado et al. (2020),^88^ who observed in thermal springs (18.5–42 °C) that its abundance declined as temperature increased. The high occurrence of *A. minutissimum* likely reflects its role as an early colonising species^89^ and its fluctuating relative abundance may reflect different stages of biofilm development. The fact that *A. minutissimum* did not dominate the community in 35 chambers by day 25 indicates that the biofilm had developed beyond the early successional stage. *F. saprophila*, commonly occurring in highly eutrophic, meso- to polysaprobic waters,^90^ did not show significant correlation with nutrients, likely due to the restricted nutrient range. Although not all species responded significantly to pesticide stress, the presence of both positive and negative associations highlights the spectrum of species-specific responses to chemical stressors.

Collectively, these candidate bioindicators respond predictably across environmentally realistic gradients, offering an early warning framework for detecting both abiotic stressors (light and temperature) and chemical disturbances (pesticides and nutrients) and supporting their potential for integration into biological indices.

In an Australian field study, Wood et al. (2019)^20^ identified a set of bioindicator taxa that differed from those reported in our study and by Morin et al. (2009).^1^ Such differences are expected, as diatom communities exhibit biogeographic structuring and are assembled from region-specific species pools.^36^ For example, several taxa found in higher abundance in herbicide-polluted sites in Australia (e.g., *Diadesmis confervacea*, *Luticola goeppertiana*, *Navicula perminuta*, *Stauroneis anceps*, and *Bacillaria paxillifera*) were not detected in our study, reflecting regional differences in species composition rather than inconsistencies in response patterns to pesticides. Differences in the identity of pesticide-associated bioindicator taxa across studies should be interpreted primarily as a consequence of biogeographic context and regional species pools, rather than as contradictory response patterns.

Light and temperature elicited the strongest species-specific responses and were the primary drivers of community-composition dissimilarities using the Bray–Curtis distance across all sequencing data: diatoms (*rbcL*), eukaryotes (*18S*) and prokaryotes (*16S*). This aligns with the hypothesis that, while overall α-diversity remained stable, community composition shifted significantly, reflecting changes in β-diversity under the experimental conditions. These results underline that light- and temperature-driven variability can confound pesticide-related effects if not controlled for.

### Drivers Moderating Functional and Structural Biofilm Responses

Biofilm tolerance to pesticides has been shown to depend on external factors, such as light intensity,^27^ temperature,^91^ and nutrient availability.^92^ The environmentally relevant exposure scenarios we employed likely moderated the stress and allowed compensatory responses, which could explain the limited functional change we observed. Adding nutrients to the relatively elevated levels in the Chriesbach water further narrowed the upper end of the experimental gradient (control treatments IDs 17–20, see Supporting Information S15), especially regarding phosphate concentrations.^53^ In parallel, the mature, dense biofilms used in this study potentially limited pesticide diffusion, as previously shown by Chaumet et al. (2019),^93^ thereby buffering the effects of the pesticides. This aligns with Guash and Sabater (1995)^94^ who found that reduced algal density increased pesticide penetration and led to stronger effects^94^.

## Limitations and Outlook

With this study, we assess whether diatoms are suitable bioindicators for pesticide stress in Swiss surface waters. The experimental setup and design were optimized to mimic field conditions, regarding realistic exposure scenarios and by integrating the diversity of exposure scenarios resulting from confounding environmental factors (nutrient concentrations, light and temperature). Although trade-offs had to be made between the goals of transferability and feasibility, which led to the following limitations, we nonetheless fulfilled the primary objective by identifying several diatom species as suitable candidate bioindicators of pesticide stress in Swiss streams. With additional field validation, these candidates could provide the basis for a diatom index targeting pesticide stress.

Candidate bioindicator taxa identified in experimental or field studies can be added into two main index families: (1) weighted-average indices based on Zelinka and Marvan formula,^95^ accounting for abundance, indicator value and specific sensitivity^96^ and (2) species-at-risk indices (SPEAR-type).^20,97^ In a weighted average indices framework, each taxon is assigned an indicator value (*D*_*i*_) representing its autecological optimum along the target gradient and a weighting factor (*G*_*i*_) reflecting its reliability as an indicator; the index is then calculated as a weighted average of *D*_*i*_ using taxon relative abundance (*H*_*i*_) and *G*_*i*_

3 $$I_{\mathrm{wa}}=\frac{\sum_{i=1}^{n}D_{i}G_{i}H_{i}}{\sum_{i=1}^{n}G_{i}H_{i}}$$

This approach retains continuous information on where a community lies along the gradient and is conceptually grounded in unimodal species responses; however, it typically requires large calibration data sets to derive stable *D*_*i*_ and *G*_*i*_ values across broad environmental ranges and under confounding conditions.^98^ By contrast, SPEAR-type indices classify taxa into vulnerable versus nonvulnerable groups based on traits (e.g., motility and herbicide sensitivity were used as traits for diatom classification in Wood et al. (2019)) and then summarize community composition as the abundance-based fraction of vulnerable taxa as

4 $$\mathrm{SPEAR}=\frac{\sum_{i=1}^{n}\;\log\left(x_{i}+1\right)y_{i}}{\sum_{i=1}^{n}\;\log\left(x_{i}+1\right)}$$

where *n* is the number of diatom species, *x*_*i*_ is the abundance of taxon *i* and *y*_*i*_ is 1 if the taxon is classified as a species at risk, or *y*_*i*_ is 0 if classified as not at risk.

This categorical structure needs less data to be robust, but it provides less quantitative resolution and depends on the availability and transferability of trait and sensitivity classifications. Consequently, while weighted average indices can be highly informative when sufficient calibration data exist, data coverage in Swiss streams may be more compatible with SPEAR-type for developing pesticide-related diatom bioindication. In practice, candidate species identified here would either be assigned quantitative sensitivity scores for a weighted average indices framework or classified into vulnerable versus non vulnerable groups for a SPEAR-type framework, based on their observed responses and trait information. A pragmatic stepwise pathway would therefore be to first expand the calibration basis through additional controlled experiments and field campaigns, and to initially implement a SPEAR-type framework, which can later be refined toward a fully parametrized weighted average indices as data coverage increases. In both cases, calibration and validation against independent field data would be required prior to operational use.

In this proof-of-concept study, biofilms were established using streamwater from a single site as the inoculum. While this approach does not capture the full diversity of diatom communities in Swiss surface waters, the occurring diatom species are representative of Swiss Plateau streams as each appears in the taxonomic list of the Swiss biomonitoring method for diatoms.^96^ The dominant taxa (see Figure 5) are also described as widespread/common in standard freshwater benthic diatom identification floras,^99^ indicating their regional representativeness. Widespread taxa are necessary for index development due to their consistent occurrence across sites;^4^ however, individual species still need to exhibit distinct responses to different stressors, as demonstrated in our experimental results. Future studies could expand this framework by incorporating inocula from multiple sites and spanning broader nutrient gradients, which would allow a more explicit disentangling of nutrient-pesticide interactions. Similarly, while we focused on a limited set of parameters (i.e., bacterial abundance, photosynthetic activity, ash-free dry weight, and chlorophyll-a) to balance feasibility and sensitivity, additional functions (e.g., respiration, extracellular enzyme activity, nutrient uptake, extracellular polymeric substances (EPS) dynamics *etc*.) could be integrated to refine the mechanistic understanding of biofilm responses.

Apart from the above-mentioned trade-offs, our results highlight the importance of medium exchange frequency, the duration of the preconditioning of the system, and the compensation for nutrient and pesticide depletion. We also recommend including replicate positive controls at the upper end of the pesticide gradient to quantify variability under high stress, where variability is expected to be lower.^100^

Our pesticide mixture stress estimates were based solely on EC_50_ values from single-species tests of algae (with diatom test not always available) and did not account for differences in measured parameters. Therefore, before the experiment it was unclear whether our target concentrations would fall within an effective range for biofilms. While an ideal approach would involve deriving biofilm-specific effect concentrations directly within the flow-chamber system, this was beyond the scope of the present study.

To summarize, the semi-randomized multifactorial design for diatom biofilms applied in this study allowed us to effectively separate environmental variables from pesticide effects while realistic mixture exposures increased the ecological relevance and transferability of the results to field conditions. Although the biofilm function remained unaffected by the tested exposure scenarios, we observed clear species-specific responses that underscore the potential of diatoms as sensitive bioindicators for pesticides. Further laboratory experiments should extend the ranges of key factors, particularly toward lower nutrient and higher pesticide concentrations to test response consistency. Analyzing existing biomonitoring data sets for correlations between pesticide stress and the abundances of species identified as pesticide-responsive would provide an independent field validation of our findings. Ultimately, advancing bioassessment requires not only technical refinements but a conceptual shift toward the acknowledgment and integration of variability and complexity of biofilm communities by critically aligning our methods with the ecological realities they aim to represent.

## Supplementary Material



## Notes

It contains detailed descriptions of the experimental setup; pesticide mixture preparation; and analytical methods; including nutrient and water quality assessments; supporting tables (experimental design showing the values of the four experimental factors (temperature, light intensity, nutrient level, and TU) for the 44 treatment IDs), figures (preparation of the exposure medium for the flow-through chamber system), and sequencing data (*rbcL*, *16S*, *18S*) for all 44 treatments. Additional sections present physicochemical monitoring; toxic-unit calculations; and taxonomic lists of identified diatoms (PDF)
