# Supplementary material for: Effects of Pesticide Mixtures and Environmental Factors on Benthic Diatom Communities: A Microcosm Approach
Source: Environ Sci Technol. 2026 Apr 20;60(17):12723–36. doi: 10.1021/acs.est.5c14415 (PMC13151046; doi:10.1021/acs.est.5c14415)
Supplement: Supplementary file 1 [file es5c14415_si_001.pdf]

Effects of pesticide mixtures and environmental factors on benthic diatom communities: a microcosm approach

Sarah Descloux[†, §], Ahmed Tlili[†], Alexandra Kroll[ǁ], Soizic Morin[#], Christoph Schür[†,‡], Kristin Schirmer[†,§], Nele Schuwirth[‡,§].*

[†]Eawag, Swiss Federal Institute of Aquatic Science and Technology, Department of Environmental Toxicology (Utox), Überlandstrasse 133, 8600 Dübendorf, Switzerland.

[‡]Eawag, Swiss Federal Institute of Aquatic Science and Technology, Department of Systems Analysis, Integrated Assessment and Modelling (Siam), Überlandstrasse 133, 8600 Dübendorf, Switzerland.

[§]ETH Zürich, Department of Environmental Systems Science, Universitätstrasse 16, 8092 Zürich, Switzerland.

[ǁ]Swiss Centre for Applied Ecotoxicology, Überlandstrasse 133, 8600 Dübendorf, Switzerland.

[#]INRAE, National Research Institute for Agriculture, Food and the Environment, UR EABX, 50 avenue de Verdun, 33612 Cestas cedex, Nouvelle-Aquitaine Bordeaux Centre, France.

*Corresponding author at: Eawag, Swiss Federal Institute of Aquatic Science and Technology, Department of Environmental Toxicology (Utox), Überlandstrasse 133, 8600 Dübendorf, Switzerland.

E-mail address: sarah.descloux@eawag.ch

This Supporting Information contains:

23 pages

8 figures (Figures S1–S8)

11 tables (Tables S1–S11)

Table of Contents

This Supporting Information contains: 2

Supplementary Section S1 Experimental setup 3

Supplementary Section S2 Treatment administration in the flow-through chamber system 4

Supplementary Section S3 Measured experimental values 5

Supplementary Section S4 Toxic unit calculation 6

Supplementary Section S5 Water quality assessment based on nutrients 8

Supplementary Section S6 Pesticide analysis and properties 10

Supplementary Section S7 Mock community algae 14

Supplementary Section S8 Sequencing data processing, ZOTU binning 15

Supplementary Section S9 Water physico-chemistry 16

Supplementary Section S10 Nutrients loss after/during exposure 18

Supplementary Section S11 List of diatom species 20

Supplementary Section S12 Diatom community sequenced using *rbcL* gene 21

Supplementary Section S13 Prokaryote community sequenced using *16S* gene 21

Supplementary Section S14 Eukaryote community sequenced using *18S* gene 22

Supplementary Section S15 Control treatment variability (IDs 17-20) 22

Supplementary Section S1 Experimental setup

The setup consisted of flow-through chambers, silicone tubing, and Schott bottles (Figure S1). The flow-through chambers were constructed by the Eawag workshop based on plans developed at Eawag in previous years. Each chamber was composed of two parallel metallic plates sealed together. One of the metallic plates featured a glass-covered opening (72 mm × 25 mm) to allow light penetration. A silicone

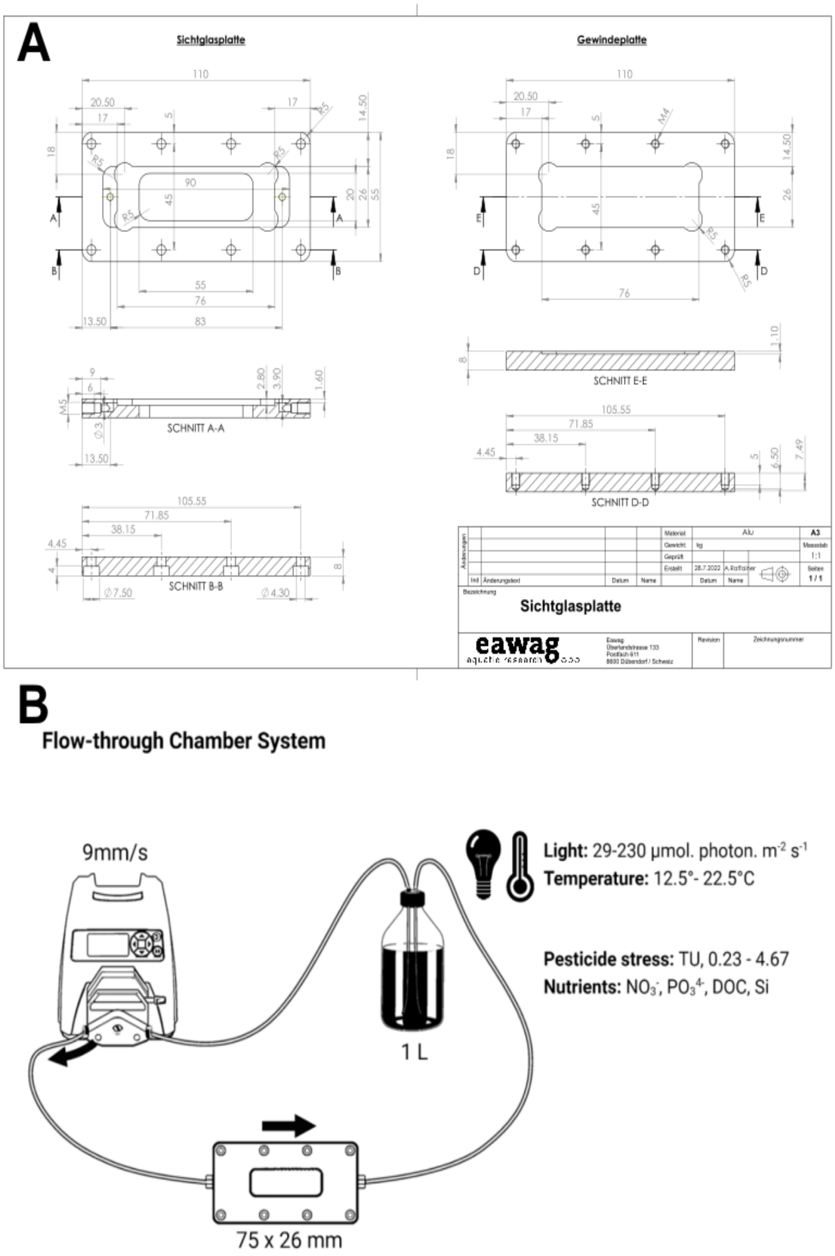

Figure S1: Illustration of the experimental setup. *Panel A shows the construction plan of the chamber plate that forms the basis of the flow-through chamber system. Panel B shows the flow-through chamber system that enables biofilm colonization, connected via silicone tubing to a peristaltic pump that supplies the closed system and one 1 L Schott bottle containing the*

layer placed between the plates ensured the system was watertight. At the center of each chamber, an empty space allowed for the placement of a microscope slide (Thermo Fisher Scientific, Waltham, MA, USA), enabling biofilm colonization. Silicone tubing (Maagtechnic, Switzerland), due to its chemical inertness and flexibility, was used to connect the system. To minimize system exposure to external factors, two holes were drilled into each Schott bottle cap using a drill press for clean and precise openings to connect the silicone tubing. Pressure-resistant, two-stop tubes (Masterflex®, Ismatec®, United States), with the maximum diameter supported by the peristaltic pump, facilitated the flow circulation. The setup was tested beforehand to ensure proper biofilm growth and to verify the system's configuration and connectivity for consistent biofilm development.

Supplementary Section S2 Treatment administration in the flow-through chamber system

To prevent chemical and nutrient depletion and waste accumulation, the medium in the closed system was replaced twice a week across all 44 treatments. Stock solutions of pesticides and nutrients were prepared separately for each treatment. During each water change, 10 µL of the corresponding

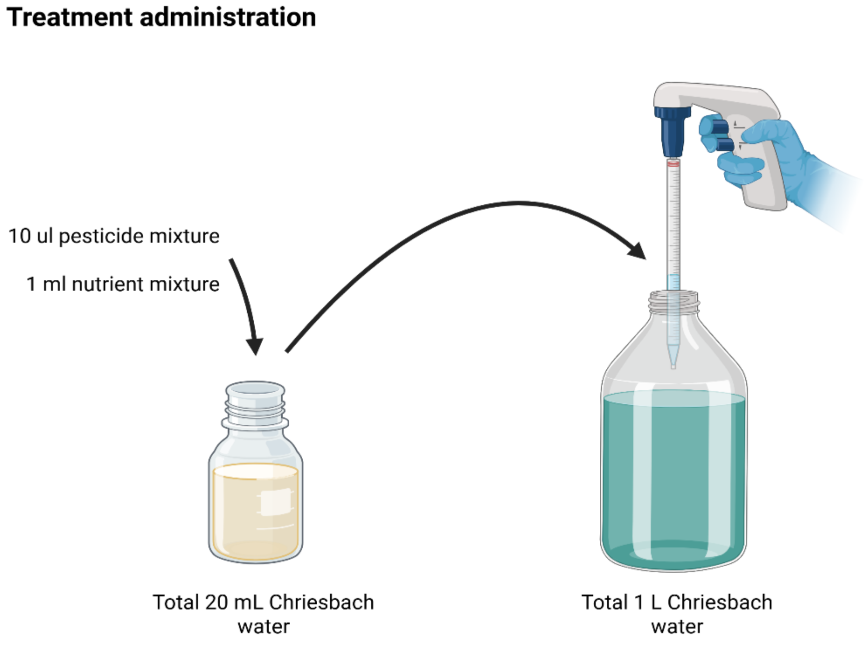

Figure S2: Preparation of the exposure medium for the flow-through chamber system. *A total of 10 µL of the pesticide mixture dissolved in methanol and 1 mL of the nutrient mixture in distilled water are first added to 20 mL of Chriesbach water to create an intermediate exposure solution. This solution is then added to 980 mL of Chriesbach water from a larger 50 L reservoir to ensure homogenization, resulting in a final volume of 1 L of medium for the experiment.*

pesticide mixture, dissolved in methanol, and 1 mL of the nutrient mixture in distilled water, were added to an intermediate exposure solution containing 20 mL of Chriesbach water to prevent localized toxicity effects (Figure S2). To ensure proper homogenization, this 20 mL intermediate exposure solution was then mixed into 980 mL of Chriesbach water medium, taken from a 50 L reservoir.

For the initial medium preparation, an additional 20 mL of biofilm suspension (optical density = 0.6), prepared from Chriesbach river biofilm, was added to promote biofilm colonization. This approach was based on preliminary tests using the same system, which visually demonstrated that the addition of biofilm suspension as an inoculum enhances biofilm colonization rates.

Supplementary Section S3 Measured experimental values

The experimental design table (Table S1) summarizes the measured experimental factors for each of our 44 treatments, detailing the actual temperature, light intensity, nutrient levels, and toxic unit (TU) values. We report average values derived from chemical analyses taken immediately before and after each exposure period.

Table S1: Experimental design showing the values of the four experimental factors (temperature, light intensity, nutrient level, and TU) for the 44 treatment IDs. *The values shown correspond to actual measurements and not the experimental design target value, as these reflect the true conditions experienced by the biofilm communities. Toxic unit and nutrient level were averaged from measurements taken before and after exposure to provide a reliable estimate of exposure.*

Treatment ID	***Temperature [°C]***	***Light intensity [µmol photons m^{-2} s^{-1}]***	***Nutrient level***	***Toxic unit***
**1**	20	74	2.23	0.13
**2**	20	74	2.15	1.24
**3**	20	74	2.86	0.16
**4**	20	74	1.95	0.10
**5**	20	230	1.67	1.73
**6**	20	230	2.22	0.47
**7**	20	230	2.26	1.79
**8**	20	230	1.73	1.96
**9**	22.5	51	2.35	0.44
**10**	22.5	51	1.21	0.08
**11**	22.5	51	1.19	1.38
**12**	22.5	51	1.85	1.84
**13**	22.5	185	2.20	1.59
**14**	22.5	185	0.92	0.85

**15**	22.5	185	2.49	0.23
**16**	22.5	185	1.74	0.91
**17**	17.5	130	0.84	0.11
**18**	17.5	130	0.85	0.22
**19**	17.5	130	0.85	0.11
**20**	17.5	130	1.02	0.16
**21**	17.5	130	3.00	0.10
**22**	17.5	130	3.02	0.06
**23**	17.5	130	2.83	0.15
**24**	17.5	130	2.86	0.16
**25**	17.5	51	1.80	3.11
**26**	17.5	51	2.48	2.09
**27**	17.5	51	2.10	0.46
**28**	17.5	51	2.81	1.42
**29**	15	29	3.06	2.38
**30**	15	29	2.72	4.25
**31**	15	29	1.03	0.94
**32**	15	29	1.66	0.25
**33**	15	163	3.11	1.34
**34**	15	163	2.89	1.44
**35**	15	163	2.66	0.37
**36**	15	163	1.56	1.48
**37**	12.5	118	2.62	0.18
**38**	12.5	118	3.01	0.16
**39**	12.5	118	1.58	1.71
**40**	12.5	118	2.80	1.68
**41**	12.5	96	2.94	0.62
**42**	12.5	96	2.10	0.17
**43**	12.5	96	1.35	3.87
**44**	12.5	96	1.46	3.48

Supplementary Section S4 Toxic unit calculation

Table S2: Pesticides EC50 from primary producers*. A total of 49 pesticides was selected based on regulations from the Federal Office of the Environment and monitored through the Swiss National Surface Water Quality Monitoring program (Micropollutants TREND). The EC50 selection was based by the EC50 values for primary producers for each pesticide.*

Pesticides	*EC50 primary producer µg/L*
**2, 4-D**	40
**Atrazine**	20.5
**Azoxystrobin**	149.1
**Bentazon**	150
**Boscalid**	1800
**Carbendazim**	7700

Pesticides	*EC50 primary producer µg/L*
**Iprovalicarb**	10000
**Isoproturon**	19
**lambda-cyhalothrin**	10000
**Linuron**	13.7
**MCPA**	64.1
**Mecoprop**	18700

**Chloridazon**	1900	**Metalaxyl**	19950
**Chlorpyrifos**	1200	**Metamitron**	390
**Chlorpyrifos-methyl**	774	**Metazachlor**	2.8
**Chlorotoluron**	24	**Methomyl**	100000
**Clothianidin**	55000	**Methoxyfenozide**	3400
**Cypermethrin**	10000	**Metolachlor**	32.6
**Cyproconazole**	59	**Metribuzin**	8.7
**Cyprodinil**	2110	**Napropamide**	680
**DEET**	41000	**Nicosulfuron**	2.3
**Diflufenican**	0.58	**Pirimicarb**	140000
**Dimethachlor**	43.4	**Propamocarb**	70400
**Dimethenamid**	25	**Propyzamid**	21
**Dimethoate**	5500	**Pyrimethanil**	5840
**Diuron**	1.4	**Spiroxamin**	6.3
**Epoxiconazole**	23.7	**Tebuconazole**	144.4
**Flufenacet**	7.55	**Terbuthylazine**	12.8
**Foramsulfuron**	0.96	**Terbutryn**	3.4
**Imidacloprid**	10000	**Thiacloprid**	96700
		**Thiamethoxam**	81800

We calculated the toxic unit (TU) for each real-world NAWA MP sample (TU_{sample}) using the script of Schuwirth (2020). For the experimental design we set targets for the TUs to achieve a good coverage of the range between 0 and 4.3 (the maximum TU_{sample}). In a second step, to get realistic pesticide mixtures, we identified real monitoring samples with a similar TU than the target. We then adjusted the concentrations of these monitoring samples proportionally to match the target TU. The selection process employed a probabilistic method that assigned a higher probability (p_{sample}) to samples more closely aligning with the TU_{target}, calculated as follows:

$$p_{sample} = \frac{1}{\Delta TU} \quad \text{Equation 1}$$

where:

$$\Delta TU = |\, TU_{target} - TU_{sample}| \quad \text{Equation 2}$$

Upon selecting a suitable sample, the concentrations of the substances measured were proportionally adjusted to precisely match the targeted TU (TU_{target}), according to the formula:

$$C_{target} = C_{sample} \times \frac{TU_{target}}{TU_{sample}} \qquad \text{Equation 3}$$

To avoid extreme mismatches, probabilities are set to zero if the TU_{sample} deviates from the TU_{target} beyond a factor defined by fd_{max}. Specifically, when the ratio TU_{target}/TU_{sample} is larger than fd_{max} or smaller than $1/fd_{max}$, the probability for choosing this sample is set to zero and the probabilities for the other samples are renormalized so that they sum up to 1. This ensures that samples with TUs too far from the target TU are excluded. If the TU_{target} is below 3, a threshold of fd_{max} = 25 is applied, while for a TU_{target} of 3 or higher, a threshold of fd_{max} = 200 is chosen. This decreased the probability of always choosing the same sample for high target TUs, because there were only few samples with TU > 1 in the monitoring data.

Deviation of the intended experimental design: Originally, we intended to have some positive control treatments with a TU of 4.3, the highest TU of the monitoring data. However, we first assumed a too small value for the EC50 of Cypermethrin of 0.1 µg/L, which was later corrected to 10000 µg/L. This resulted in the positive control not reaching the intended maximum TU. However, the realized range still captures environmentally relevant toxic units (from 0 to 4).

Supplementary Section S5 Water quality assessment based on nutrients

The range of the target nutrient values was set by two conditions: (1) no addition, where 0 mg/L represented no added nutrients, reflecting the baseline levels in the Chriesbach water, and (2) maximum concentrations determined by FOEN classifications for a bad ecological status of watercourses (Table S6). For phosphate, the maximum value was doubled to account for the high concentrations observed across Swiss monitoring programs, particularly in rivers influenced by lakes.

As silicic acid does not have specific regulatory thresholds under FOEN classifications, we based our target levels on data from Swiss monitoring programs, using the 90th percentile of the highest recorded concentrations. The target values for nutrient additions were based on measured nutrient concentrations in Chriesbach by subtracting the measured baseline concentration from the target values of the experimental design.

Nutrient concentrations were measured weekly throughout the experiment. Measurements were taken for all treatments at the start of the experiment and for a random subset of five treatments before each water change in the fresh medium to ensure consistent starting values. After each water change, all 44 treatments were measured to calculate the realized nutrient levels.

Nutrient concentrations are interpolated from predefined ranges using the following linear approximation:

$$C_{nutrient} = C_{min} + \left(\frac{level_{target} - level_{min}}{level_{max} - level_{min}}\right) \cdot (C_{max} - C_{min}) \qquad \text{Equation 6}$$

To determine the final realized concentrations for each treatment, the mean concentration before water changes was calculated and averaged with the mean of the weekly measurements after water changes. These calculated means were translated into nutrient categories, allowing us to evaluate how closely the realized nutrient levels aligned with the target concentrations.

*Table S3: **Chemical status classes for nutrients in Swiss surface waters**. They are defined by the Federal Office for the Environment (FOEN) (Liechti, 2010).*

Assessment	Ortho-P (mg P L⁻¹)	Nitrate (mg N L⁻¹)	Dissolved Organic Carbon (DOC) (mg C L⁻¹)
Very good	≤ 0.02	≤ 1.5	≤ 2.0
Good	0.02 - < 0.04	1.5 - < 5.6	2.0 - < 4.0
Moderate	0.04 - < 0.06	5.6 - < 8.4	4.0 - < 6.0
Poor	0.06 - < 0.08	8.4 - < 11.2	6 - < 8.0
Bad	≥ 0.08	≥ 11.2	≥ 8.0

Supplementary Section S6 Pesticide analysis and properties

A panel of 49 pesticides was quantified by HPLC-MS/MS using a Thermo UltiMate 3000 coupled to a PAL HTS-xt autosampler and an Atlantis T3 column (3 × 150 mm, 3 µm) at 300 µL/min and 30 °C. Electrospray ionization (±4 kV, 325 °C) was performed first on an LTQ Orbitrap XL (140 000 resolution, data-dependent acquisition) and subsequently on a Q Exactive Orbitrap for targeted MS/MS with inclusion lists. All high-resolution data were processed in Skyline software, and the accompanying table shows each compound's limit of quantification (LOQ) (Table S4).

*Table S4: **Limits of quantification (LOQ, µg per injection) for the 49 pesticide compounds.** They are determined by HPLC-MS/MS using Atlantis T3 chromatography and Orbitrap mass spectrometry.*

Pesticide	*LOQ [ug]*
**2 4-D**	0.02109375
**Atrazin**	5.74E-04
**Azoxystrobin**	2.18
**Bentazon**	0.0684833
**Boscalid**	0.265625
**Carbendazim**	8.25E-04
**Chloridazon**	0.875855

Pesticide	*LOQ [ug]*
**Isoproturon**	1.0945
**Linuron**	0.019555
**MCPA**	0.003906
**Mecoprop**	0.013916
**Metalaxyl**	0.00198
**Metamitron**	3.7875
**Metamitron-Desamino**	0.0188

**Chlorpyrifos**	0.03905
**Chlorpyrifos-methyl**	0.03905
**Chlortoluron**	6.978423333
**Clothianidin**	0.0037116
**Cypermethrin**	0.7
**Cyproconazol**	0.00723
**Cyprodinil**	1.01
**DEET**	0.00352
**Diflufenican**	0.01409
**Dimethachlor**	0.01075
**Dimethoat**	9.52E-04
**Diuron**	0.00433
**Epoxiconazol**	0.0391
**Flufenacet**	0.216
**Foramsulfuron**	0.02345
**Imidacloprid**	0.019793333

**Metazachlor**	0.00732
**Methoxyfenozid**	0.010548
**Metolachlor**	0.281
**Metribuzin**	0.00557
**Napropamid**	0.488
**Nicosulfuron**	0.01875
**Pirimicarb**	0.005275
**Propamocarb**	0.6033
**Propyzamide**	0.02013
**Pyrimethanil**	9.53E-04
**Spiroxamin**	0.14065
**Tebuconazol**	0.192
**Terbuthylazin**	0.0381
**Terbutryn**	0.0258
**Thiacloprid**	5.86E-04
**Thiamethoxam**	1.601565

Given the broad spectrum of log Kow values across our 49 pesticides, from highly hydrophilic (log Kow < 0) to strongly hydrophobic (>5), we pre-conditioned the silicone tubing by using each pesticide mixture and aiming saturation in the system before the actual experiment (Table S5). This "tubing pre-exposure" step minimizes initial sorption losses to the system tubing and helps maintain intended treatment concentrations, thereby ensuring a more consistent, steady pesticide exposure throughout the experiment.

Table S5: **List of log Kow values for the 49 pesticides used, categorized into three groups**. Compounds with higher log Kow values are more likely to adsorb onto surfaces, while those with lower values remain more soluble in water.

Pesticides	***log Kow***
**2-4-D (Dichlorophenoxyacetic acid)**	2.62
**Atrazin**	2.61
**Azoxystrobin**	2.5
**Bentazon**	2.34
**Boscalid**	2.96
**Carbendazim**	1.52
**Chloridazon**	1.2
**Chlorpyrifos**	4.7
**Chlorpyrifos-methyl**	4.31
**Chlortoluron**	2.41
**Clothianidin**	1.12
**Cypermethrin**	6.6
**Cyproconazol**	2.9
**Cyprodinil**	3.99
**DEET**	2.4
**Diflufenican**	4.2
**dimethachlore**	2.17
**Dimethenamid**	1.89
**Dimethoat**	0.78
**Diuron**	2.87
**Epoxiconazol**	3.44
**Flufenacet**	3.5
**Foramsulfuron**	0.6
**Imidacloprid**	0.57
**Iprovalicarb**	3.2

Pesticides	***log Kow***
**Isoproturon**	2.87
**lambda-Cyhalothrin**	5.5
**Linuron**	3
**MCPA**	2.52
**Mecoprop**	2.94
**Metalaxyl**	1.71
**Metamitron**	1.44
**Metazachlor**	2.49
**methomyl**	0.6
**Methoxyfenozid**	3.72
**Metolachlor**	3.13
**Metribuzin**	1.7
**Napropamid**	3.36
**Nicosulfuron**	-1.77
**Pirimicarb**	1.7
**Propamocarb**	-0.4
**Propyzamid**	3.27
**Pyrimethanil**	2.84
**Spiroxamin**	2.79
**Tebuconazol**	3.7
**Terbuthylazin**	3.4
**Terbutryn**	3.74
**Thiacloprid**	0.73
**Thiamethoxam**	-0.13

Table S6: Hydrolysis half-lives. *Compounds covering the lowest and highest log Kow values: Napropamide (3.36), Terbutylazine (4.00), Terbutryn (3.74), Chloridazon (1.20), Metamitron (1.44), Nicosulfuron (−1.77), Atrazine (2.61), Diuron (2.87), Diflufenican (4.20), Bentazon (2.34), Foramsulfuron (0.60), Isoproturon (2.87).*

Pesticides	***half-hydrolysis_[days]PPDB***	***half-hydrolysis_[days]Ecotox_center***
**Napropamide**	Stable	Stable
**Terbuthylazine**	Stable	>200
**Terbutryn**	Stable	-
**Chloridazon**	Stable	>30
**Metamitron**	480	pH 7 = 479.6 days, pH 9 = 8.5 days
**Nicosulfuron**	Stable	>32
**Atrazine**	86	
**Diuron**	8.8	
**Diflufenican**	133	
**Bentazon**	Stable	
**Foramsulfuron**	128	
**Isoproturon**	1560	

Table S7: ***Henry's law constants (k_h) for pesticides***. *Values are reported in the solubility form k_h [mol m^{-3} Pa^{-1}] at ~20–25 °C.*

Pesticides	*Henry law_constant [mol/(m3Pa)]*	*Pesticides*	*Henry law_constant [mol/(m3Pa)]*
**2-4-D**	4.0×10^{-06}	**Isoproturon**	8.8×10^{4}
**Atrazine**	1.9×10^{3}	**Linuron**	1.6×10^{3}
**Azoxystrobin**	1.4×10^{8}	**MCPA**	7.4×10^{3}
**Bentazon**	4.5×10^{3}	**Mecoprop**	1.1×10^{4}
**Boscalid**	1.9×10^{4}	**Metalaxyl**	3.3×10^{3}
**Carbendazim**	4.7×10^{5}	**Metamitron**	1.0×10^{6}
**Chlorpyrifos**	1.8	**Metazachlor**	1.3×10^{1}
**Chlortoluron**	7.0×10^{4}	**Methomyl**	2.0×10^{2}
**Clothianidin**	3.4×10^{10}	**Methoxyfenozide**	2.6×10^{6}
**Cypermethrin**	1	**Metolachlor**	7.5×10^{2}
**Cyproconazole**	1.4×10^{4}	**Metribuzin**	8.2×10^{4}
**Cyprodinil**	1.2×10^{2}	**Napropamide**	1.2×10^{4}
**DEET**	4.7×10^{2}	**Nicosulfuron**	6.8×10^{10}
**Diflufenican**	6.4×10^{1}	**Pirimicarb**	1.2×10^{4}
**Dimethachlor**	5.9×10^{3}	**Propamocarb**	2.1×10^{3}
**Dimethenamid**	8.9×10^{2}	**Propyzamid**	1.0×10^{3}
**Dimethoate**	4.1×10^{4}	**Pyrimethanil**	3.4×10^{2}
**Diuron**	3.5×10^{1}	**Spiroxamin**	4.0×10^{2}
**Flufenacet**	1.7×10^{3}	**Tebuconazol**	7.0×10^{4}
**Foramsulfuron**	1.7×10^{11}	**Terbuthylazin**	4.3×10^{2}
**Imidacloprid**	4.9×10^{9}	**Terbutryn**	4.7×10^{2}
**Iprovalicarb**	7.1×10^{5}	**Thiacloprid**	9.0×10^{8}
		**Thiamethoxam**	2.1×10^{9}

Supplementary Section S7 Mock community algae

A total of 27 photosynthetic microorganisms was included as a positive-control community, comprising 11 green algae (Chlorophyta), 12 diatoms (Bacillariophyta), and 4 cyanobacteria (Cyanobacteria), ensuring that our sequencing workflow reliably amplifies representatives across all three major lineages.

Table S8: Positive-control community composition*. 11 green algae (Chlorophyta), 12 diatoms (Bacillariophyta), and 4 cyanobacteria (Cyanobacteria).*

	Species
**1**	*Stigeoclonium aestivale*

**2**	*Oedogonium sp*
**3**	*Ulothrix mucosa*
**4**	*Botryococcus braunii*
**5**	*Spirogyra sp*
**6**	*Pediastrum duplex*
**7**	*Pediastrum boryanum*
**8**	*Navicula cuspidata (Craticula cuspidata)*
**9**	*Cymbella cistula*
**10**	*Fragilaria cf. Capucina*
**11**	*Nitzschia vermicularis*
**12**	*Synedra sp.*
**13**	*Tabellaria sp.*
**14**	*Achnanthidium pyrenaicum*
**15**	*Navicula accomoda (Craticula accomoda)*
**16**	*Sellaphora nigri*
**17**	*Ulnaria ulna*
**18**	*Gomphonema clavatum*
**19**	*Chamaesiphon polonicus*
**20**	*Merismopedia glauca*
**21**	*Pseudanabaena galeata*
**22**	*Phormidium sp.*
**23**	*Mougotia sp*
**24**	*Scenedesmus acuminatus*
**25**	*Scenedesmus vacuolatus*
**26**	*Chlorella vulgaris*
**27**	*Fragilaria crotonensis*

Supplementary Section S8 Sequencing data processing, ZOTU binning

The reads were processed using USEARCH v11.0.667_i86 linux64 for all datasets. End trimming was performed with fastx_truncate (rbcL: R1/R2 = 20/40; 16S: R1/R2 = 25/50; 18S: R1/R2 = 0/0). Read pairs were merged using fastq_mergepairs with dataset-specific parameters (minimum overlap: 16S = 30 bp, rbcL = 30 bp, 18S = 15 bp; minimum identity = 60%). Primers were removed using search pcr (amplicon size range: 100–600 bp; mismatches: 16S/18S = 1, rbcL = 2). Quality filtering was conducted with PRINSEQ-lite v0.20.4 (minimum Q mean = 20, dust complexity = 30), with size and GC ranges adjusted for each dataset (rbcL: 200–300 bp, GC = 30–70%; 16S: 350–450 bp, GC = 30–70%; 18S: 400–600 bp, GC = 20–80%). ZOTUs were generated using UNOISE3 for exact sequence variant detection, and

additional clustering was performed at 99%, 98%, and 97% identity levels where required. Taxonomic assignment was conducted using SINTAX with confidence thresholds of 0.85, referencing the SILVA SSU v138 database (16S), PR2 SSU database (18S), and INRAE rbcL database v92 (rbcL).

Supplementary Section S9 Water physico-chemistry

Illustration of the temporal stability of water-quality parameters, conductivity, pH, and dissolved oxygen, across our 44 treatments. Measurements were made immediately before the first water change in the fresh medium (28 August 2023) and then twice after water renewal, i.e. after 5 days and 25 days of exposure (1 September and 21 September 2023). Conductivity, pH, and O_2 concentrations remained relatively constant over the three sampling times, demonstrating that our twice-weekly water changes effectively maintained physicochemical conditions within narrow bounds throughout the experiment and across treatments (Table S9 & Figure S3).

Table S9: Water physico-chemistry over time, showing mean values with standard deviations for conductivity (Cond σ), pH, and dissolved oxygen (O_2 mg/L) across three measurement dates. *Measurements were taken before the water change in the fresh medium (28.08.2023) and after the water change (01.09.2023 and 21.09.2023) to assess variations in water quality parameters. Minimum and maximum values are also indicated.*

Date	*Time*	*Parameters*	*Mean*	*Sd*	*Min*	*Max*
**28.08.2023**	before	Cond (σ)	600.7	49.65	511	675
**01.09.2023**	after	Cond (σ)	611.84	54.13	523	708
**21.09.2023**	after	Cond (σ)	629.66	61.55	522	798
**28.08.2023**	before	pH	8.02	0.13	7.8	8.4
**01.09.2023**	after	pH	8.16	0.14	8	8.6
**21.09.2023**	after	pH	8.06	0.1	7.89	8.34
**28.08.2023**	before	O_2 (mg/L)	8.74	0.3	8.12	9.19
**01.09.2023**	after	O_2 (mg/L)	8.81	0.28	8.22	9.24
**21.09.2023**	after	O_2 (mg/L)	8.77	0.34	8.12	9.23

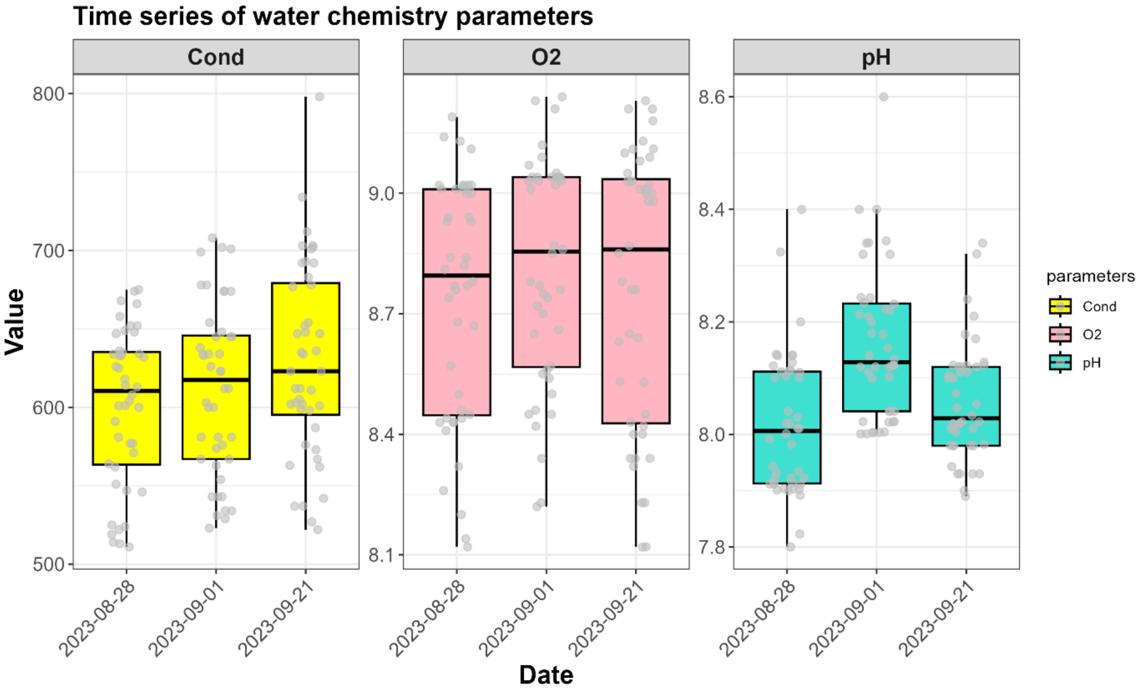

Figure S3: Water physico-chemistry over time, showing mean values with standard deviations for conductivity (Cond σ), pH, and dissolved oxygen (O_2 mg/L) across three measurement dates. *Measurements were taken before (28.08.2023) and after the water change (01.09.2023 and 21.09.2023) to assess variations in water quality parameters. Each grey dot represents one treatment.*

Supplementary Section S10 Nutrients loss after/during exposure

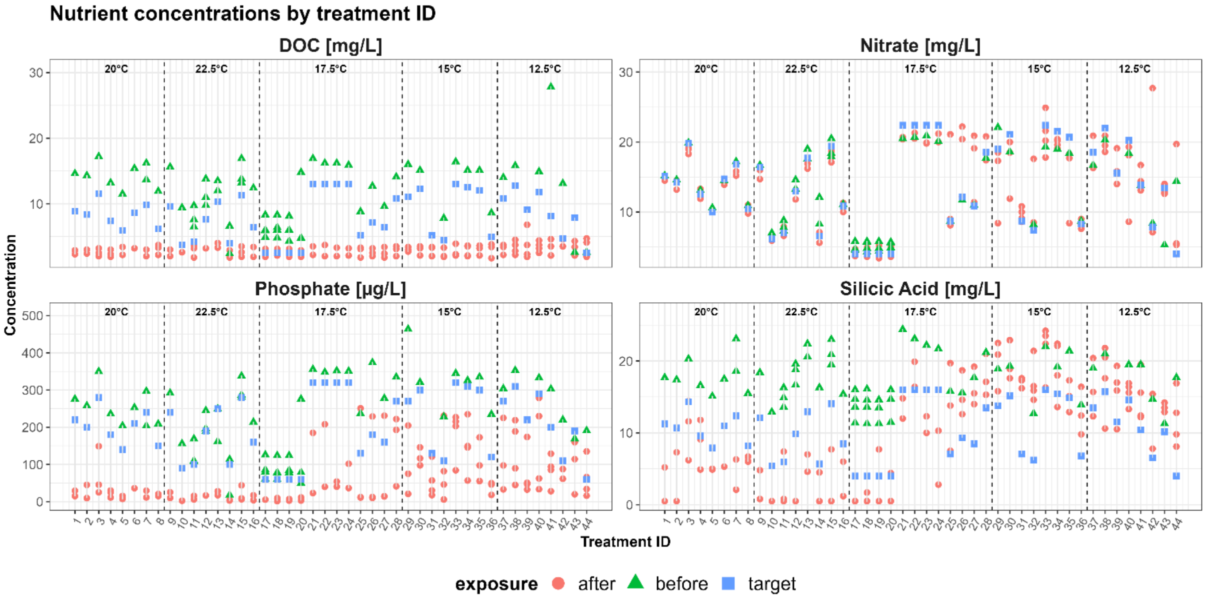

Figure S4: Nutrient concentrations, dissolved organic carbon (DOC), nitrate (NO_3^-), phosphate (PO_4^{3-}), and silicic acid ($HSiO_4$) across 44 treatment conditions. *The y-axis represents nutrient concentrations (mg/L for DOC, nitrate, and silicic acid; µg/L for phosphate), while the x-axis displays individual samples or treatments. Red circles indicate water nutrient concentration after exposure, green triangles represent nutrient measurements in the fresh medium immediately at the administration of the treatments, and blue squares show the target nutrient values.*

Dynamics of four nutrients (nitrate, phosphate, silicic acid, and dissolved organic carbon (DOC)) over exposure and the course of the experiment (Figure S4). Phosphate, silicic acid, and DOC generally declined markedly over each 3–4 day of exposure interval, confirming rapid uptake and/or

transformation in the flow-through chambers. Notably, DOC and phosphate exhibited the steepest drops, whereas nitrate showed no or a more moderate reduction.

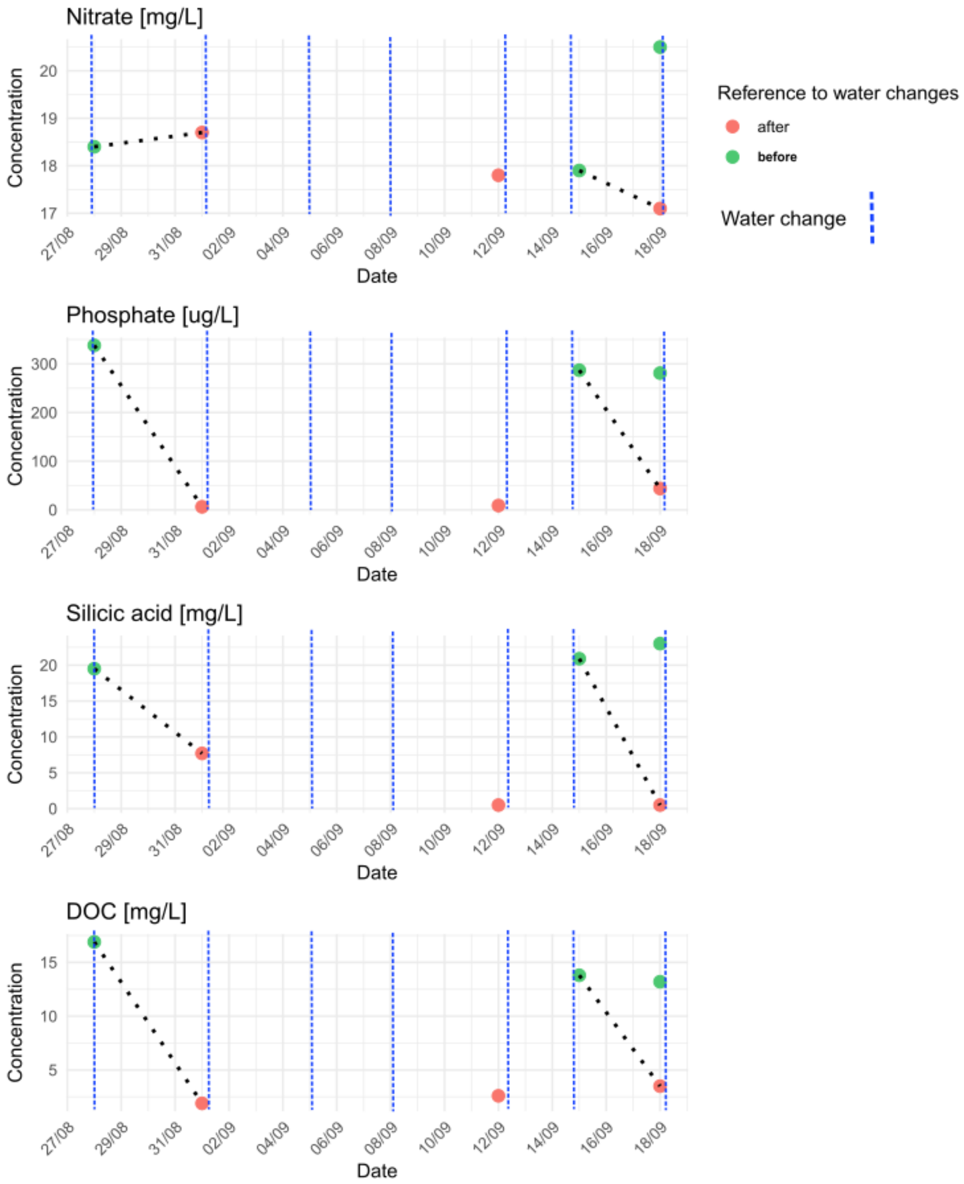

__Figure S5: This figure shows four panels, each corresponding to a different nutrient, Nitrate mg/L, Phosphate µg/L, Silicic acid mg/L, and DOC mg/L, with the x-axis representing experiment dates.__ In each panel, green circles denote "before" measurements taken immediately after the administration of the treatments at the beginning of the exposure period, and red circles indicate "after" measurements taken at the end of the exposure period. Dotted black lines illustrates how concentrations vary between these two points in time. Dotted blue lines represent the water change moment, happening twice a week.

Supplementary Section S11 List of diatom species

A total of 50 diatom species were identified by sequencing the rbcL gene.

Table S10: Diatom species identified using Next-Generation Sequencing (NGS).

	Species
**1**	*Achnanthidium minutissimum*
**2**	*Amphora copulata*
**3**	*Amphora ovalis*
**4**	*Amphora pediculus*
**5**	*Caloneis fontinalis*
**6**	*Caloneis sp*
**7**	*Conticribra weissflogii*
**8**	*Craticula buderi*
**9**	*Craticula molestiformis*
**10**	*Cyclotella cryptica*
**11**	*Cyclotella meneghiniana*
**12**	*Diatoma tenuis*
**13**	*Encyonema ventricosum*
**14**	*Eunotia arcus*
**15**	*Fistulifera saprophila*
**16**	*Fragilaria gracilis*
**17**	*Gomphonella olivacea*
**18**	*Gomphonema saprophilum*
**19**	*Karayevia ploenensis var gessneri*
**20**	*Mayamaea permitis*
**21**	*Melosira varians*
**22**	*Navicula antonii*
**23**	*Navicula cryptocephala*
**24**	*Navicula cryptotenella*
**25**	*Navicula gregaria*

	Species
**26**	*Navicula tripunctata*
**27**	*Navicula trivialis*
**28**	*Navicula veneta*
**29**	*Nitzschia amphibia*
**30**	*Nitzschia capitellata*
**31**	*Nitzschia cf. acicularoides*
**32**	*Nitzschia cf. microcephala*
**33**	*Nitzschia cf. pusilla*
**34**	*Nitzschia dissipata var media*
**35**	*Nitzschia fonticola*
**36**	*Nitzschia linearis*
**37**	*Nitzschia palea*
**38**	*Nitzschia paleacea*
**39**	*Nitzschia supralitorea*
**40**	*Planothidium frequentissimum*
**41**	*Planothidium victori*
**42**	*Sellaphora nigri*
**43**	*Sellaphora pupula*
**44**	*Sellaphora saugerresii*
**45**	*Sellaphora seminulum*
**46**	*Staurosira venter*
**47**	*Tryblionella apiculata*
**48**	*Tryblionella sp*
**49**	*Ulnaria acus*
**50**	*Ulnaria ulna*

Supplementary Section S12 Diatom community sequenced using *rbcL* gene

Synthesis of distance-based and variance-based multivariate analyses.

*Table S11: Summary of multivariate analyses evaluating the effects of light, temperature, nutrients, and pesticide exposure (TU) on diatom community structure. Effect sizes are reported as R^2 values for distance-based PERMANOVA (Bray–Curtis, Jaccard, and cosine distance) and as adjusted R^2 values for redundancy analysis (RDA) based on Hellinger-transformed community data. Significance levels are indicated as * $p < 0.05$, ** $p < 0.01$, *** $p < 0.001$.*

Method	***Test***	***Light***	***Temperature***	***Nutrients***	***TU***
Bray-Curtis	PERMANOVA	0.107 ***	0.237 ***	0.023 ns	0.011 ns
Jaccard	PERMANOVA	0.067 **	0.063 **	0.028 ns	0.026 ns
Hellinger	RDA	0.033 ***	0.078 ***	0.008 ns	0.005 ns
Cosine	PERMANOVA	0.113 **	0.321 ***	0.024 ns	0.005 ns

Supplementary Section S13 Prokaryote community sequenced using *16S* gene

Non-metric multidimensional scaling (NMDS) ordination of 16S rRNA gene–based prokaryotic community composition across our experimental factors.

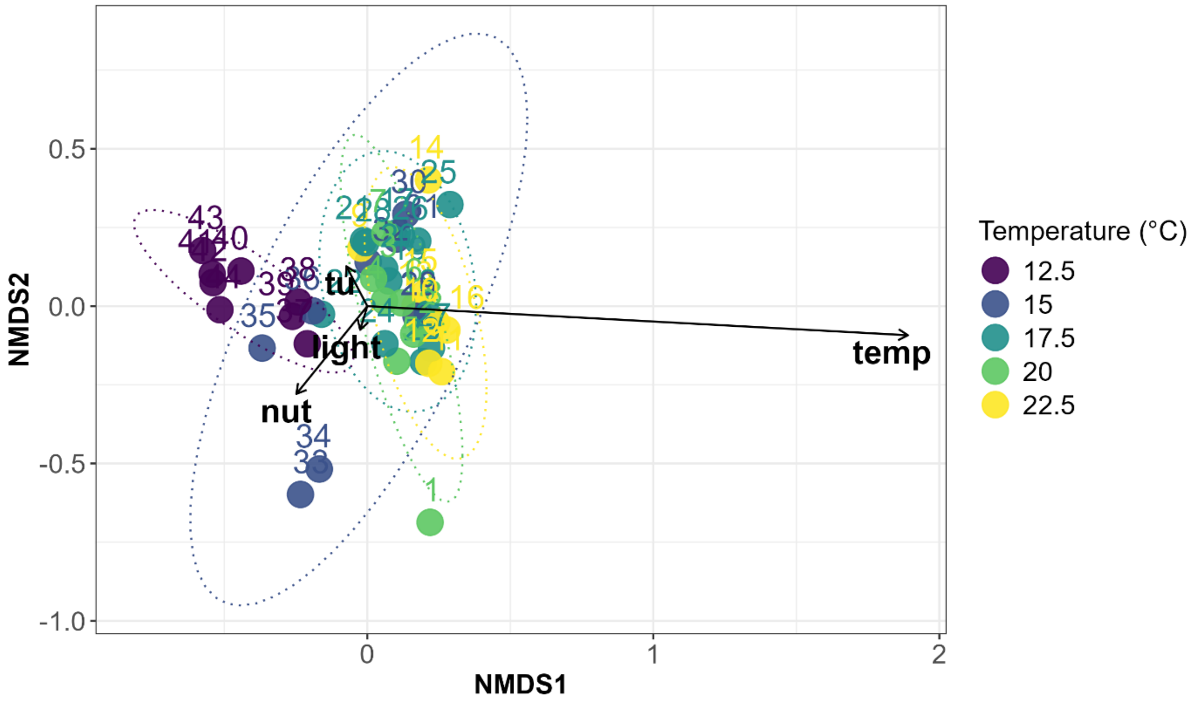

Figure S6: NMDS ordination (k = 2, stress = 0.057) of 16S rRNA gene–based prokaryote community composition at the phylum (Bray–Curtis dissimilarity). Samples are colored by temperature treatment (°C) and enclosed by 95% confidence ellipses around treatment centroids. *PERMANOVA (adonis2) on Bray–Curtis distances revealed a highly significant effect of temperature ($R^2 = 0.591$, $p = 0.001$), whereas light ($R^2 = 0.025$, $p = 0.125$), nutrient concentration ($R^2 = 0.117$, $p = 0.189$) and toxic-unit exposure ($R^2 = 0.046$, $p = 0.608$) were not significant.*

Supplementary Section S14 Eukaryote community sequenced using *18S* gene

Non-metric multidimensional scaling (NMDS) ordination of 18S rRNA gene–based eukaryotic community composition across our experimental factors.

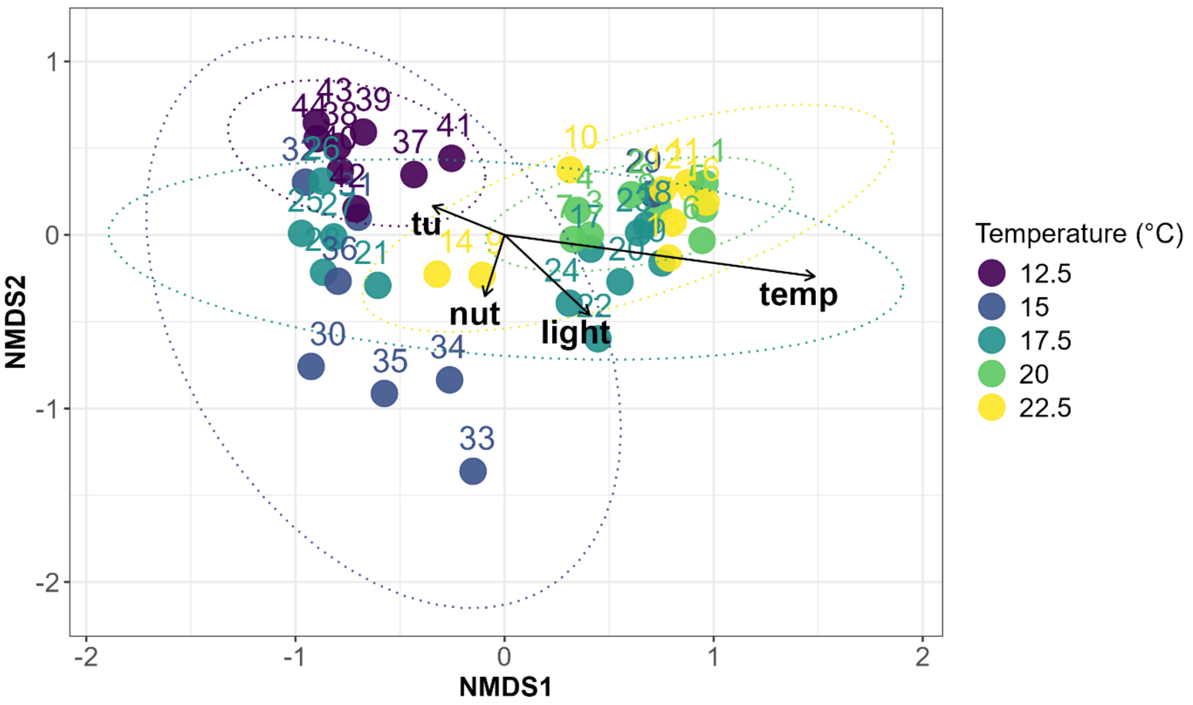

Figure S7: NMDS ordination (k = 2, stress = 0.13) of 18S rRNA gene–based eukaryotic community composition at the genus level using Bray–Curtis dissimilarity. Points are colored by temperature treatment (°C) and 95 % confidence ellipses are drawn around each temperature centroid. *PERMANOVA (adonis2, by = "margin") on Bray–Curtis distances showed that temperature explained a highly significant fraction of the variance ($R^2 = 0.218$, $p = 0.001$), light intensity had a smaller but significant effect ($R^2 = 0.069$, $p = 0.012$), whereas nutrient concentration ($R^2 = 0.028$, $p = 0.116$) and toxic-unit exposure ($R^2 = 0.031$, $p = 0.077$) were not significant*

Supplementary Section S15 Control treatment variability (IDs 17-20)

The control treatments included four replicates (IDs 17-20). Each replicate was evaluated for ash-free dry weight (AFDW), bacterial cell counts, chlorophyll-a content normalized to AFDW and photosynthetic efficiency.

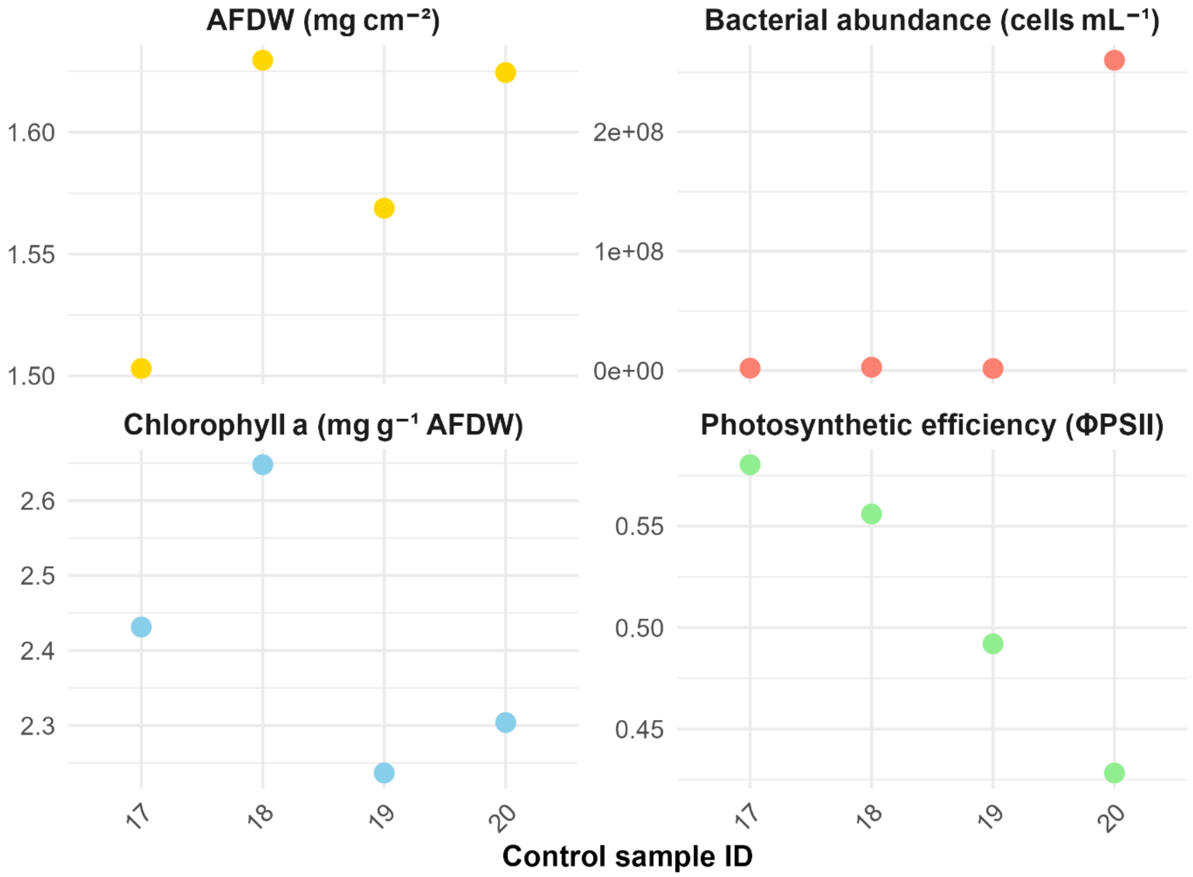

Figure S 8: ***Control treatment (IDs 17–20).*** *Functional and structural biofilm descriptors measured during the experiment.*
